# Inhibition of autophagy curtails visual loss in a model of autosomal dominant optic atrophy

**DOI:** 10.1038/s41467-020-17821-1

**Published:** 2020-08-12

**Authors:** Marta Zaninello, Konstantinos Palikaras, Deborah Naon, Keiko Iwata, Stephanie Herkenne, Ruben Quintana-Cabrera, Martina Semenzato, Francesca Grespi, Fred N. Ross-Cisneros, Valerio Carelli, Alfredo A. Sadun, Nektarios Tavernarakis, Luca Scorrano

**Affiliations:** 1 Veneto Institute of Molecular Medicine, Via Orus 2, Padova, Italy; 2 Department of Biology, University of Padova, Via U. Bassi 58B, Padova, Italy; 3 IRCCS Fondazione Santa Lucia, Via Ardeatina 306, Rome, Italy; 4 Institute of Molecular Biology and Biotechnology, Foundation for Research and Technology-Hellas, Heraklion, Crete Greece; 5 Department of Basic Sciences, Faculty of Medicine, University of Crete, Heraklion, Crete Greece; 6 Doheny Eye Institute, Los Angeles, CA USA; 7 IRCCS Institute of Neurological Sciences of Bologna, Bellaria Hospital, Bologna, Italy; 8 Unit of Neurology, Department of Biomedical and Neuromotor Sciences (DIBINEM), University of Bologna, Bologna, Italy; 9 Department of Ophthalmology, David Geffen School of Medicine at UCLA, Los Angeles, CA USA

**Keywords:** Mechanisms of disease, Autophagosomes, Mitochondria

## Abstract

In autosomal dominant optic atrophy (ADOA), caused by mutations in the mitochondrial cristae biogenesis and fusion protein optic atrophy 1 (Opa1), retinal ganglion cell (RGC) dysfunction and visual loss occur by unknown mechanisms. Here, we show a role for autophagy in ADOA pathogenesis. In RGCs expressing mutated Opa1, active 5’ AMP-activated protein kinase (AMPK) and its autophagy effector ULK1 accumulate at axonal hillocks. This AMPK activation triggers localized hillock autophagosome accumulation and mitophagy, ultimately resulting in reduced axonal mitochondrial content that is restored by genetic inhibition of AMPK and autophagy. In *C. elegans*, deletion of AMPK or of key autophagy and mitophagy genes normalizes the axonal mitochondrial content that is reduced upon mitochondrial dysfunction. In conditional, RGC specific *Opa1*-deficient mice, depletion of the essential autophagy gene *Atg7* normalizes the excess autophagy and corrects the visual defects caused by *Opa1* ablation. Thus, our data identify AMPK and autophagy as targetable components of ADOA pathogenesis.

## Abstract (web summary)

Autosomal dominant optic atrophy is caused by mutations in the mitochondrial fusion protein OPA1. Here, the authors show that AMPK-induced autophagy depletes mitochondria in axons of retinal ganglion cells and that autophagic inhibition reverses vision loss in a mouse model.

## Introduction

Polarized cells like neurons are particularly susceptible to defects in mitochondrial fusion or fission. Fusion events are controlled by the outer mitochondrial membrane (OMM) mitofusin (Mfn) 1 and 2^1,2^ and the inner mitochondrial membrane (IMM) optic atrophy 1 (Opa1)^3–5^. Fission depends on dynamin-related protein 1 (Drp1) and on its OMM adaptors Fission 1 (Fis1)^6^, mitochondrial fission factor (Mff)^7^, and mitochondrial dynamics proteins (Mid) 49 and 51^8^. Genetic diseases caused by Drp1, Mfn2, and Opa1 mutations primarily lead to neurological manifestations^9^. When these mitochondria-shaping proteins are mutated, mitochondria are dysfunctional: ATP production is not sustained, and mitochondria consume it by the reversal of the ATP synthase in attempt to maintain their membrane potential^10^. ATP consumption by damaged mitochondria increases AMP levels leading to AMPK activation that eventually reinforces mitochondrial fragmentation and causes autophagy^11,12^.

Autosomal dominant optic atrophy (ADOA) is an untreatable disease caused by *OPA1* mutations^13,14^. ADOA is clinically characterized by early childhood bilateral visual loss and the cells primarily affected by the disease are RGCs^15,16^. However, due to the limited accessibility of retinal material from patients, most of our current knowledge on ADOA pathogenesis comes from studies in rodent models. Changes in autophagy are prominent features of mouse models of ADOA. In primary neurons and RGCs, expression of mutated *Opa1* not only recapitulates the mitochondrial morphological defects observed in other cellular models, but also reduces dendritic mitochondrial content^17,18^. It also leads to the accumulation of autophagosomes in vitro and in vivo^19,20^. This latter feature is perhaps not surprising, considering that hallmarks of autophagy often accompany neuronal mitochondrial damage and that autophagy has been linked to axonal and dendritic dysfunction^21–24^. However, it is unclear whether activation of autophagy is per se a trigger of neurodegeneration caused by impaired mitochondrial function, or a compensatory response of the stressed RGC^25^. This question is patent in the context of ADOA, whose pathobiology is unclear and perhaps cannot be ascribed to the established roles of Opa1 in mitochondrial fusion, cristae biogenesis, apoptosis, and metabolism^26^.

We therefore set out to explore the mechanism by which mutated Opa1 impairs RGC function and causes visual loss. Opa1 mutations or deletion cause the accumulation of autophagosomes at the axonal hillock and reduced axonal mitochondrial content in primary RGCs. Genetic inhibition of AMPK, of autophagy and of mitophagy restores axonal mitochondrial content. This mechanism is also conserved in neurons of the nematode *Caenorhabditis elegans* that are similarly depleted of mitochondria upon mitochondrial dysfunction. In vivo, autophagy inhibition prevents the autophagosome accumulation and the loss of vision in a mouse model of ADOA generated by targeted RGCs *Opa1* deletion.

## Results

### Localized mitophagy in proximity of the axonal hillock of ADOA RGCs

**Fig. 1:**
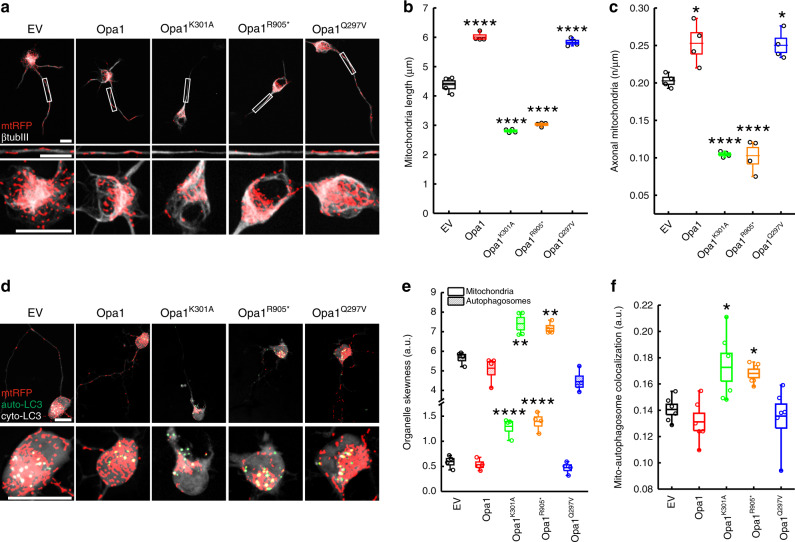
Mitochondrial content is reduced in axons of ADOA RGCs. **a** Representative z-projections of stacks of confocal images of mtRFP (red) in primary RGCs co-transfected with the indicated plasmids and after 24 h fixed and immunostained with β-tubulin III (βtub III, grey). Axons and somas were magnified in the bottom panels. EV, empty vector. Bars, 20 μm. **b** Quantification of mitochondrial length from four independent experiments as in (**a**) (EV, *n* = 86; Opa1, *n* = 113; Opa1^K301A^, *n* = 82; Opa1^R905*^, *n* = 71; Opa1^Q297V^, *n* = 114 cells). ****p < 0.0001 vs. EV in one-way ANOVA/Tukey’s test. **c** Quantification of mitochondrial content in axons from four independent experiments as in (**a**) (EV, *n* = 58; Opa1, *n* = 65; Opa1^K301A^, *n* = 69; Opa1^R905*^, *n* = 54; Opa1^Q297V^, *n* = 64 cells). *p = 0.018 Opa1, *p* = 0.027 Opa1^Q297V^ vs. EV; ****p < 0.0001 vs. EV in one-way ANOVA/Bonferroni’s test. **d** Representative z-projections of stacks of confocal images acquired 24 h after transfection of primary RGCs co-transfected with mtRFP (red) and YFP-LC3 (green, autophagosome-LC3, auto-LC3) and the indicated plasmids. The cytoplasmic YFP-LC3 signal (cyto-LC3) is pseudocolored in grey for the sake of clarity. The region corresponding to the soma was magnified in the inset. Bars, 20 μm. **e** Quantification of soma mitochondria and autophagosome distribution towards the axonal hillock in 4 independent experiments as in (**d**) (EV, *n* = 65 mitochondria and 49 autophagosomes; Opa1, *n* = 69 mitochondria and autophagosomes; Opa1^K301A^, *n* = 58 mitochondria and 47 autophagosomes; Opa1^R905*^, *n* = 69 mitochondria and autophagosomes; Opa1^Q297V^, *n* = 70 mitochondria and autophagosomes) **p = 0.0023 Opa1^K301A^ vs. EV; *p* = 0.0084 Opa1^R905*^ vs. EV; ****p < 0.0001 vs. EV in one-way ANOVA/Tukey’s test. **f** Quantification of mitochondria and autophagosome co-localization in six independent experiments as in (**d**). (EV, *n* = 86; Opa1, *n* = 86; Opa1^K301A^, *n* = 101; Opa1^R905*^, *n* = 82; Opa1^Q297V^, *n* = 91 cells). *p = 0.0153 Opa1^K301A^, *p* = 0.043 Opa1^R905*^ vs. EV in one-way ANOVA/Tukey’s test. In box plots, centre line represents mean, bounds of boxes SEM, whiskers the 10th–90th percentiles; each dot represents independent experiments. Source data are provided as a Source Data file.

The role of Opa1 in mitochondrial function has been extensively investigated in multiple organs and organisms including tissues of neuronal origin, but the pathogenic mechanism of ADOA is still unclear^26^. Therefore, we decided to investigate the effect of the expression of mutated Opa1 in RGCs, the cell type primarily affected by the disease. Because the GTPase and the coiled-coil domains of *OPA1* are hotspots of ADOA mutations^27^, we used two mouse *Opa1* mutants modeling frequent ADOA mutations in these two regions: Opa1^K301A^, a GTPase domain mutant that reduces Opa1 GTPase activity^28^, and Opa1^R905*^, a truncative mutant of the coiled-coil domain important for Opa1 protein-protein interaction^27^. In addition, as a control mutant we expressed Opa1^Q297V^, a constitutively active form of Opa1 that is not an ADOA mutant^29^. Indeed, while Opa1^K301A^ and Opa1^R905*^ fragment mitochondria and induce Opa1-dependent apoptosis^4,28,30^, Opa1^Q297V^ shows opposite effects^29^. First, we verified the effect of Opa1 mutants on mitochondrial morphology and function in enriched cultures of primary RGCs from mouse retinas^31^. RGCs were electroporated and the efficiency of transfection was validated by OPA1 immunostaining: all vectors were expressed at the same level because the intensities of the OPA1 fluorescence were comparable (Supplementary Fig. 1a, b). As expected given the known pro-fusion activity of Opa1, Opa1 and Opa1^Q297V^ coexpressed with mitochondrial targeted dsRED (mtRFP) triggered mitochondrial elongation, while the pathogenic ADOA mutants Opa1^K301A^ and Opa1^R905*^ shortened mitochondria in axons (Fig. 1a, b). These data recapitulated findings already known from other models^4,28–30^. Interestingly, axons of pathogenic mutants were also depleted of mitochondria (Fig. 1a, c). This observation was similar to what is shown in dendrites of cortical neurons^18^ and to what we measured in Cre-transduced Opa1^fl/fl^ RGCs^32^ (Supplementary Fig. 1c, d). The observed reduction in axonal density of mitochondria was not a consequence of mtRFP fluorescence dimming. Indeed, we recorded a similar reduction in axonal mitochondrial density when we immunostained mitochondria using the OMM marker TOM20 in Opa1^K301A^ RGCs (Supplementary Fig. 1e, f). Moreover, fragmented mitochondria appeared at different distances from the soma (Fig. 1a, c).

**Fig. 2:**
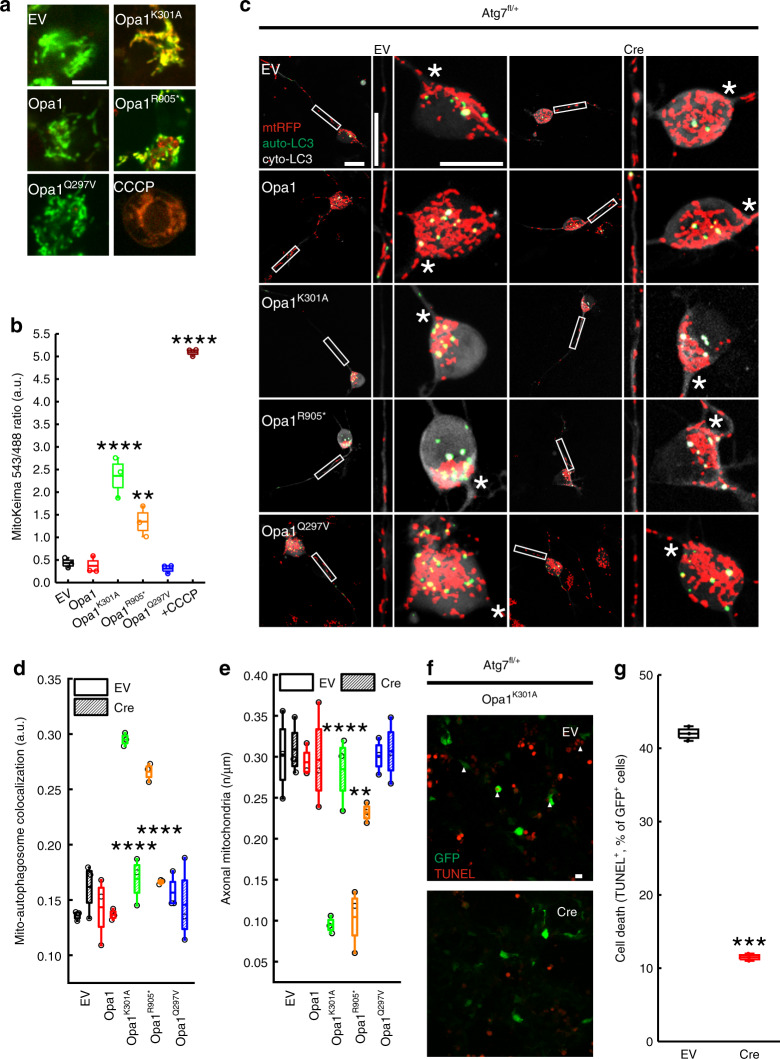
Inhibition of autophagy restores mitochondrial content in ADOA RGCs axons. **a** Representative z-projections of stacks of confocal images of mitoKeima fluorescence in RGCs 24 h after cotransfection with the indicated plasmids. Where indicated, empty vector (EV) cotransfected cells were treated for 2 h with carbonyl cyanide m-chlorophenylhydrazone (CCCP). Bar, 10 µm. **b** MitoKeima ratio from three independent experiments as in (**a**) (EV, *n* = 60; Opa1, *n* = 60; Opa1^K301A^, *n* = 56; Opa1^R905*^, *n* = 60; Opa1^Q297V^, *n* = 59; EV + CCCP, *n* = 58 fields) **p = 0.079; ****p < 0.0001 vs. EV in one-way ANOVA/Tukey’s test. **c** Representative z-projections of stacks of confocal images of mtRFP (red) and YFP-LC3 (green, autophagosome-LC3, auto-LC3) fluorescence in primary Atg7^fl/+^ RGCs co-transfected as indicated. The cytoplasmic YFP-LC3 signal (cyto-LC3) is pseudocolored in grey for the sake of clarity. Boxed axonal regions and the soma region were magnified in the corresponding bottom panels. Asterisks: axonal hillocks. Bars, 20 μm. **d** Mitochondria-autophagosomes co-localization from three independent experiments as in (**c**) (EV: EV, *n* = 54; Opa1, *n* = 54; Opa1^K301A^, *n* = 50; Opa1^R905*^, *n* = 45; Opa1^Q297V^, *n* = 58 cells; Cre: EV, *n* = 45; Opa1, *n* = 57; Opa1^K301A^, *n* = 54; Opa1^R905*^, *n* = 51; Opa1^Q297V^, *n* = 54 cells). ****p < 0.0001 vs. EV in two-way ANOVA/Sidak’s test. **e** Mitochondrial content in axons from three independent experiments as in (**c**) (EV: EV, *n* = 54; Opa1, *n* = 54; Opa1^K301A^, *n* = 52; Opa1^R905*^, *n* = 53; Opa1^Q297V^, *n* = 57 cells; Cre: EV, *n* = 55; Opa1, *n* = 56; Opa1^K301A^, *n* = 55; Opa1^R905*^, *n* = 55; Opa1^Q297V^, *n* = 54 cells). ****p < 0.0001; **p = 0.003 vs. EV in two-way ANOVA/Sidak’s test. **f** Representative confocal images of Atg7^fl/+^ RGCs co-transfected with GFP (green) and the indicated plasmids and processed for TUNEL (red) after 24 h. Arrowheads: GFP^+^, TUNEL^+^ cells. Scale bar, 10 µm. **g** Percentage of TUNEL positivity from three independent experiments as in (**f**) (EV, *n* = 139; Cre, *n* = 168 GFP^+^ cells). ***p = 0.0002 vs. EV in parametric *t* test/Welch’s test. In box plots, centre line represents mean, bounds of boxes SEM, whiskers the 10th–90th percentiles; each dot represents independent experiments. Source data are provided as a Source Data file.

Fragmentation of mitochondria is a prerequisite of mitophagy^25^ and autophagic structures have been identified by electron microscopy in somas of RGCs from ADOA mouse models^19,20^. We therefore tested if autophagy was altered in vitro in the RGC model of ADOA by labeling autophagic vesicles with a yellow fluorescent protein (YFP)-LC3 sensor. In ADOA RGCs, mitochondria and YFP-LC3^+^ vesicles mostly accumulated close to the axonal hillock and were almost always absent from axons (Fig. 1d, e). Expression of the lipidation deficient GFP-LC3^G120A^ mutant in RGCs excluded that these LC3^+^ structures represented aggregates due to overexpression of the fluorescent marker (Supplementary Fig. 1g)^33^. Colocalization between autophagic vesicles and mitochondria was 25–30% higher in ADOA RGCs compared to control-transfected RGCs, suggesting that ADOA mitochondria could be targeted for autophagic degradation (Fig. 1d, f). Indeed, the specific mitophagy probe mitoKeima^34^ recorded a 1.5- to 2.5-fold increase in mitophagy in ADOA RGCs (Fig. 2a, b). Thus, mitochondria are targeted for autophagic degradation close to hillocks of ADOA RGCs.

### Autophagy inhibition corrects axonal mitochondrial content in ADOA RGCs

In neurons, the degradation of damaged mitochondria in axons has been proposed to occur by different mechanisms. Some studies pointed to the soma as the site of degradation following retrograde transport of damaged mitochondria^35,36^. Alternatively, mitochondria can be degraded distally in axons^37^. In RGCs, we rarely retrieved autophagosomes in wt and ADOA axons (Figs. 1d and 2c). Conversely, we recorded increased mitophagy in the soma (Fig. 2a, b). Altogether, these data suggest that the reduced mitochondrial content in the axon of ADOA RGCs could be due to mitochondrial degradation by autophagy in the soma. To test this hypothesis we genetically curtailed autophagy in RGCs. The autophagy gene *Atg7* regulates autophagosome elongation^38^ and its genetic downregulation is frequently used to manipulate autophagic flux^39^. We therefore turned to RGCs purified from *Atg7*^fl/+^ mice^39^. Cre expression in *Atg7*^fl/+^ RGCs resulted in the expected downregulation of *Atg7* levels (Supplementary Fig. 2a, b). In *Atg7*^fl/+^ RGCs autophagic flux was inhibited, as indicated by the accumulation of LC3^+^ vesicles and of the autophagosome cargo p62 when autophagosome degradation was inhibited by bafilomycin or autophagosome formation was induced by rapamycin (Supplementary Fig. 2c–f). Having established that autophagic flux is curtailed in *Atg7*^-/+^ RGCs, we measured whether genetic autophagy inhibition affected the changes in mitochondria-autophagosome co-localization in the soma and in axon mitochondrial content caused by expression of the pathogenic Opa1 mutants. When we expressed these mutants together with Cre in *Atg7*^fl/+^ RGCs, mitochondria and autophagosomes did not colocalize and axonal mitochondrial content was not reduced (Fig. 2c–e), suggesting that in RGCs degradation of mitochondria in the soma regulates the abundance of mitochondria in the axon. Finally, when expressed in primary RGCs Opa1^K30A^ and Opa1^R905*^ lead to apoptosis, as already observed in mouse embryonic fibroblasts^30^ (Fig. 2f, g; Supplementary Fig. 3a, b). On the other hand, when we co-expressed these pathogenic Opa1 mutants with Cre in *Atg7*^fl/+^ RGCs, apoptosis was curtailed (Fig. 2g). In conclusion, autophagy inhibition restores axonal mitochondrial density in ADOA RGCs and reduces death of RGCs expressing ADOA pathogenic mutants.

### Mutated Opa1 causes local hillock AMPK activation

**Fig. 3:**
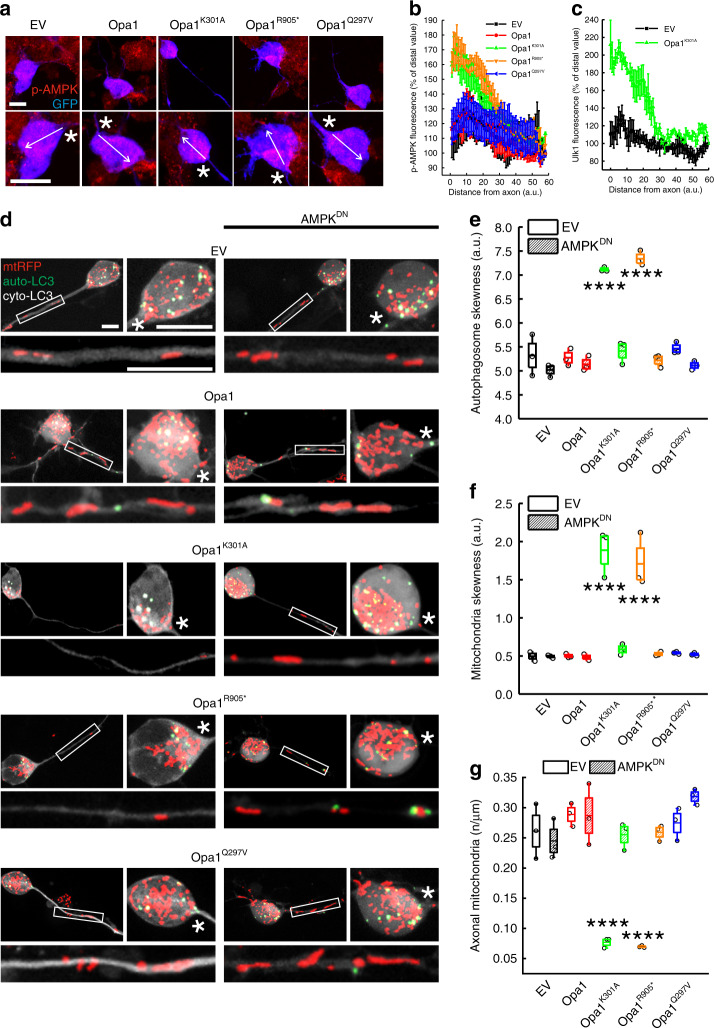
Peri-hillock AMPK activation reduces axonal mitochondrial content in ADOA RGCs. **a** Representative z-projections of stacks of confocal images of phosphorylated-AMPK (p-AMPK, red) and GFP (blue) fluorescence in primary RGCs co-transfected as indicated and after 24 h immunostained for p-AMPK. The vector from the axonal hillock to the opposite side of the soma indicates the line used to measure p-AMPK fluorescence intensity in (**b**). Asterisks: axon. Bars, 20 μm. **b** Average ± SEM of p-AMPK fluorescence intensity along the vector drawn in the soma of RGCs co-transfected with GFP and the indicated plasmids in four independent experiments as in (**a**). EV, *n* = 73; Opa1, *n* = 69; Opa1^K301A^, *n* = 75; Opa1^R905*^, *n* = 73; Opa1^Q297V^, *n* = 71 cells. **c** Average ± SEM ULK1 fluorescence intensity along the vector drawn in the soma of RGCs co-transfected with GFP and the indicated plasmids in three independent experiments as in (**a**) except that cells where immunostained for ULK1. EV, *n* = 21; Opa1^K301A^, *n* = 26 cells. **d** Representative z-projections of stacks of confocal images of mtRFP (red) and YFP-LC3 (green, autophagosome-LC3, auto-LC3) in primary RGCs co-transfected as indicated. The cytoplasmic YFP-LC3 signal (cyto-LC3) is pseudocolored in grey for the sake of clarity. Boxed axonal regions and the soma region were magnified in the corresponding bottom panels. Bars, 20 μm. **e**, **f** Quantification of autophagosomes (**e**) and mitochondria (**f**) distribution in somas from three independent experiments as in (**d**). In (**e**), for EV: EV, *n* = 54; Opa1, *n* = 53; Opa1^K301A^, *n* = 51; Opa1^R905*^, *n* = 52; Opa1^Q297V^, *n* = 52 cells; for AMPK^DN^: EV, *n* = 46; Opa1, *n* = 48; Opa1^K301A^, *n* = 60; Opa1^R905*^, *n* = 55; Opa1^Q297V^, *n* = 51 cells. In (**f**) for EV: EV, *n* = 54; Opa1, *n* = 53; Opa1^K301A^, *n* = 51; Opa1^R905*^, *n* = 51; Opa1^Q297V^, *n* = 52 cells; for AMPK^DN^: EV, *n* = 46; Opa1, *n* = 48; Opa1^K301A^, *n* = 60; Opa1^R905*^, *n* = 55; Opa1^Q297V^, *n* = 51 cells. ****p < 0.0001 vs. EV in two-way ANOVA/Sidak’s test. **g** Quantification of mitochondrial content in RGCs axons from three independent experiments as in (**d**). For EV: EV, *n* = 53; Opa1, *n* = 46; Opa1^K301A^, *n* = 51; Opa1^R905*^, *n* = 47; Opa1^Q297V^, *n* = 52 cells; for AMPK^DN^: EV, *n* = 59; Opa1, *n* = 53; Opa1^K301A^, *n* = 59; Opa1^R905*^, *n* = 50; Opa1^Q297V^, *n* = 52 cells. ****p < 0.0001 vs. EV in two-way ANOVA/Sidak’s test. In box plots, centre line represents mean, bounds of boxes SEM, whiskers the 10th–90th percentiles; each dot represents independent experiments. Source data are provided as a Source Data file.

We next investigated if the autophagosomes accumulation close to the axon resulted from a local activation of autophagy in this region. The AMP-activated protein kinase (AMPK) is a natural candidate to trigger autophagy and mitophagy in response to mitochondrial dysfunction^40–42^. When the AMP/ATP ratio increases, AMPK is activated by phosphorylation at Thr172 (p-AMPK), increasing its kinase activity^43^. We therefore investigated subcellular localization of p-AMPK in ADOA RGCs. When we immunostained these cells for p-AMPK, we found it accumulated proximal to the axon (Fig. 3a, b). p-AMPK can phosphorylate ULK1, driving its translocation to mitochondria and mitophagy^40,41^. When we immunostained RGCs expressing Opa1^K301A^ for ULK1, we similarly found it accumulated at the hillock (Fig. 3c). Because these data place AMPK activation in the region where mitochondria and autophagosomes congregate and where we measured mitophagy, we posited a role for AMPK in the mitochondria-autophagosome colocalization and in the reduction of axonal mitochondrial content observed in ADOA RGCs. To verify this hypothesis, we turned to experiments of genetic AMPK blockage, by employing a dominant negative AMPK^T172A^ mutant^43^ (AMPK^DN^). We confirmed that AMPK^DN^ reduces autophagic flux^44^ in RGCs (Supplementary Fig. 4a–c). The increased mitochondria–autophagosomes colocalization caused by the expression of these ADOA Opa1 mutants was curtailed when we coexpressed them with AMPK^DN^ (Supplementary Fig. 4d). AMPK^DN^ also restored the uniform mitochondria and autophagosomes distribution in the soma (Fig. 3d–f) as well as the mitochondrial content in the axons of ADOA RGCs (Fig. 3d, g). Thus, AMPK^DN^ not only curtails autophagosome accumulation but also restores mitochondrial distribution in ADOA RGCs. Taken together, these results place AMPK in the signaling cascade triggered by mutated Opa1 in RGCs.

### AMPK-dependent regulation of mitochondrial axonal content in *C. elegans*

**Fig. 4:**
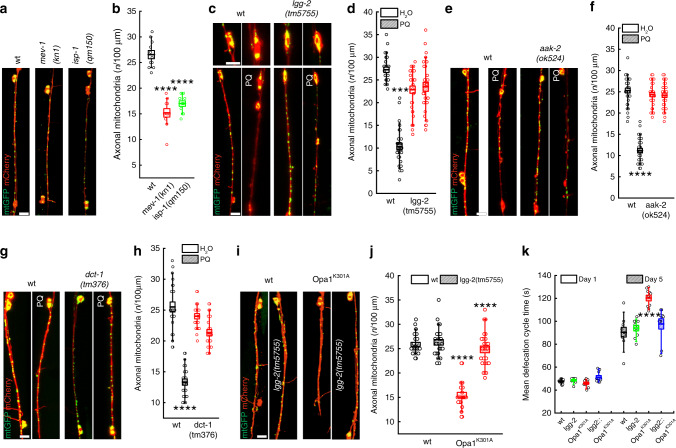
Inhibition of autophagy restores mitochondrial content in axons of *C. elegans* carrying mitochondrial dysfunction. **a** Confocal images of mtGFP (green) and cytoplasmic mCherry (red) in GABA motor neurons of the indicated transgenic nematodes. Bar, 20 μm. **b** Quantification of mitochondrial content in axons in *n* = 13 animals from three independent experiments as in (**a**). ****p < 0.0001 in one-way ANOVA/Tukey’s test. **c** Confocal images of mtGFP (green) and cytoplasmic mCherry (red) in GABA motor neurons of transgenic nematodes treated as indicated. Upper panels: magnified soma region. PQ, paraquat. Bars, 20 μm. **d** Quantification of mitochondrial content in axons in *n* = 25 animals from three independent experiments as in (**c**). **p = 0.0012 in one-way ANOVA/Tukey’s test. **e** Confocal images of mtGFP (green) and cytoplasmic mCherry (red) in GABA motor neurons of transgenic nematodes treated as indicated. Bar, 20 μm. **f** Quantification of mitochondrial content in axons in *n* = 25 animals from three independent experiments as in (**e**). ****p < 0.0001 in one-way ANOVA/Tukey’s test. **g** Confocal images of mtGFP (green) and cytoplasmic mCherry (red) in GABA motor neurons of transgenic nematodes treated as indicated. Bar, 20 μm. **h** Quantification of mitochondrial content in axons in *n* = 25 animals from three independent experiments as in (**g**). ****p < 0.0001 in one-way ANOVA/Tukey’s test. **i** Confocal images of mtGFP (green) and cytoplasmic mCherry (red) in GABAergic motor neurons of the indicated transgenic nematodes expressing Opa1^K301A^. Bar, 20 μm. **j** Quantification of mitochondrial content in axons in *n* = 25 animals from three independent experiments as in (**i**). ****p < 0.0001 in one-way ANOVA/Tukey’s test. **k** Quantification of defecation motor program duration recorded in *n* = 10 transgenic nematodes from three independent experiments at the indicated days of life. ****p < 0.0001 in one-way ANOVA/Tukey test. In box plots, centre line represents mean, bounds of boxes SEM, whiskers the 10th–90th percentiles; each dot represents an individual nematode form the indicated *N* of independent experiments. Source data are provided as a Source Data file.

The nematode *C. elegans* allows to combine in vivo imaging with genetics. It is therefore a suitable model to address whether autophagosome accumulation controls axonal mitochondrial density in vivo. We first verified if mitochondrial dysfunction led to a reduction in axon mitochondrial content in worms co-expressing a mitochondrially targeted green fluorescent protein (mtGFP) and a cytoplasmic monomeric Cherry fluorescent protein (mCherry) in GABAergic motor neurons. Several mitochondria were imaged in the axons of these transgenic nematodes. Conversely, axonal mitochondrial density was reduced by ~45% in worms deficient for mitochondrial complex II (MEV-1) or complex III (ISP-1) components (Fig. 4a, b) and by ~60% in worms treated with the mitochondrial poison paraquat^45^ (Fig. 4c, d). In transgenic worms expressing GFP-tagged LGG-2 to label autophagosomes, we observed mitochondria–autophagosome co-localization close to axons upon paraquat treatment (Supplementary Fig. 5a). Moreover, using the sensor Rosella targeted to mitochondria we specifically measured increased mitophagy in paraquat treated worms (Supplementary Fig. 5b). Like in ADOA RGCs, genetic inhibition of autophagy/mitophagy by depletion of the Atg8 homologue LGG-2, of the nematode AMPK homologue AAK-2 or of the worm mitophagy gene DCT-1/Nix restored axonal mitochondrial density in neurons of paraquat-treated nematodes (Fig. 4c–h). These experiments indicate that in *C. elegans* the axonal content of mitochondria is regulated by the same autophagy-related mechanism described in mouse RGCs.

We next decided to generate a worm model of ADOA by expressing *Opa1*^*K301A*^ in *C. elegans* GABAergic motor neurons. Also in *C. elegans* GABAergic axons, expression of *Opa1*^*K301A*^ reduced mitochondrial content in axons that was restored by genetic autophagy inhibition (Fig. 4i, j). These experiments substantiate in a different neuron type and species the pathogenic mechanism triggered by mutated Opa1 in primary mouse RGCs. Interestingly, in aged Opa1^K301A^ transgenic nematodes the defecation cycle time, a proxy of GABAergic function^46^, was prolonged by ~30% and normalized by depletion of the essential autophagy component LGG-2/Atg8 (Fig. 4k), suggesting that autophagy inhibition might exert a protective role also on complex neuronal function in ADOA models.

### Autophagy inhibition corrects visual loss of an RGC-specific *Opa1* knockout mouse

**Fig. 5:**
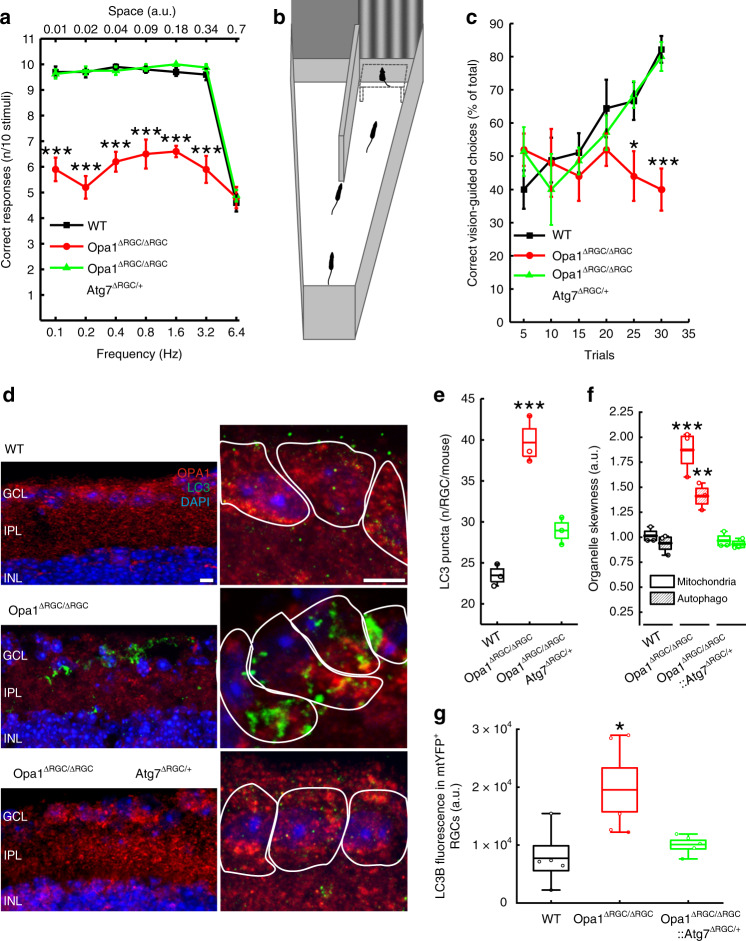
Genetic autophagy inhibition curtails the visual defect of an ADOA mouse model. **a** Average ± SEM visual acuity of 4-month-old mice of the indicated genotype in the optokinetic test. Mice were subjected to visual stimuli at the indicated temporal and spatial frequencies. Visual acuity is proportional to the percentage of correct answers to the stimulus. WT, *n* = 10; Opa1^ΔRGC/ΔRGC^, *n* = 10; Opa1^ΔRGC/ΔRGC^Atg7^ΔRGC/+^, *n* = 8 mice. ****p < 0.0001 vs. WT in two-way ANOVA/Tukey’s test. **b** A schematic of the Y-shaped pool used for the vision guided forced-swimming test. The two screens (gray and vertically striped) used as visual cues and the submerged platform are shown. **c** Average ± SEM visual acuity of 4-month-old mice of the indicated genotype in the vision guided forced-swimming test. WT, *n* = 9; Opa1^ΔRGC/ΔRGC^, *n* = 5; Opa1^ΔRGC/ΔRGC^Atg7^ΔRGC/+^, *n* = 7 mice. *p = 0.05, ***p = 0.0001 vs. WT in two-way ANOVA/Tukey test. **d** Representative z-projections of stacks of confocal images of the fluorescence of OPA1 (red), LC3B (green), and DAPI (blue) of retinal slices of 4-month-old mice of the indicated genotype. The soma of RGCs are circled with white lines in right panels. Bars, 20 μm. GCL ganglion cell layer, IPL inner plexiform layer, INL inner nuclear layer. **e** Quantification of RGCs autophagosomes number in three independent experiments as in (**d**). WT, *n* = 163; Opa1^ΔRGC/ΔRGC^, *n* = 155; Opa1^ΔRGC/ΔRGC^Atg7^ΔRGC/+^, *n* = 156 cells. ***p = 0.0002 vs. WT in one-way ANOVA/Bonferroni’s test. **f** Quantification of mitochondria and autophagosomes accumulation in axonal hillocks of RGCs in three independent experiments as in (**d**). For mitochondria, WT, *n* = 165; Opa1^ΔRGC/ΔRGC^, *n* = 155; Opa1^ΔRGC/ΔRGC^Atg7^ΔRGC/+^, *n* = 156; for autophagosomes, WT, *n* = 164; Opa1^ΔRGC/ΔRGC^, *n* = 155; Opa1^ΔRGC/ΔRGC^Atg7^ΔRGC/+^, *n* = 155 cells. **p = 0.003 ***p = 0.001 vs. WT in one-way ANOVA/Tukey’s test. **g** Quantification of anti-LC3B antibody signal in sorted mtYFP^+^ RGCs from five animals of each indicated genotype. *p = 0.012 vs. WT in one-way ANOVA/Kruskal–Walli’s test. In box plots, centre line represents mean, bounds of boxes SEM, whiskers the 10^th^–90^th^ percentiles; each dot represents an individual nematode form the indicated *N* of independent experiments. Source data are provided as a Source Data file.

To test whether autophagy inhibition could modify the natural history of ADOA in vivo, we first decided to develop an ADOA mouse model based on conditional *Opa1* ablation in RGCs. To this end we crossed *Opa1*^fl/fl^ with Grik4-Cre mice that express the Cre recombinase exclusively in RGCs^47^, thus avoiding unwanted influence from deletion of *Opa1* also in other retinal cells. Moreover, Grik4^+^ RGCs represents a RGCs subpopulation sensitive to moving stimuli^47,48^, i.e., the ones presented to the mice in the behavioural tests performed in our study. The resulting *Opa1*^fl/fl^::Grik4-Cre (*Opa1*^ΔRGC/ΔRGC^) mice were viable, fertile and no abnormal phenotype was observed at necropsy. We traced the cells in which recombination occurred by crossing Grik4-Cre mice with a Lox-Stop-Lox-mtYFP (*mtYFP*^flstop/flstop^) reporter mouse line^49^. The Cre-dependent recombination should generate only a subset of RGCs which are mtYFP^+^ cells. Indeed, mtYFP^+^ cells sorted by flow cytometry represented ~1% of total retinal cells and were positive for the RGC marker Brn3a (Supplementary Fig. 6a, b). As expected, mtYFP^+^ cells represented a subpopulation of RGCs because other Brn3a^+^ cells were mtYFP^−^ (Supplementary Fig. 6a). We applied the same tracing strategy to identify the Opa1-deficient RGCs in *Opa1*^ΔRGC/ΔRGC^ mice. We therefore further crossed *Opa1*^ΔRGC/ΔRGC^ with mtYFP^flstop/flstop^ mice and purified mtYFP^+^ RGCs by flow cytometry. In these RGCs traced for recombination, *Opa1* mRNA levels were reduced by ~50% (Supplementary Fig. 6c, d). We next tested the impact of this *Opa1* deletion on visual acuity. In a classic optokinetic assay, visual acuity was normal at every spatial and temporal frequency tested in 3 months old *Opa1*^ΔRGC/ΔRGC^ mice (Supplementary Fig. 6e). Conversely, starting from 4 months *Opa1*^ΔRGC/ΔRGC^ mice developed a ~50% decrease in visual acuity (Fig. 5a) that was sustained until we monitored mouse vision (12 months of age; Supplementary Fig. 6f–m). The visual defect was confirmed in a second test based on vision-guided swimming choice to a submerged platform in a Y-shaped pool (Fig. 5b), in which 4-month-old *Opa1*^ΔRGC/ΔRGC^ mice failed to identify the platform positioned below the visual cue (Fig. 5c and Movies 1 and 2). The results of these behavioral tests demonstrated the onset of visual defects in 4-month-old animals. To verify if the mouse model was mimicking the mitochondrial and autophagosomal phenotype observed in vitro, we measured the levels of LC3^+^ vesicles and the distribution of these organelles and of mitochondria in *Opa1*-deleted RGCs in vivo. Immunostaining of retinal sections revealed that mitochondria and autophagosomes were distributed asymmetrically in *Opa1*-deleted RGCs in mice (Fig. 5d, f). Furthermore, the abundance of LC3^+^ puncta per cell was doubled in retinal sections of ADOA mice (Fig. 5d, e) and the intensity of LC3B fluorescence was also doubled in sorted mtYFP+ RGCs by FACS (Fig. 5g). These data indicate that like in RGCs expressing Opa1 mutants, autophagosomes accumulate and mitochondria and autophagosomes are asymmetrically distributed in *Opa1*-deficient RGCs in vivo. Since inhibition of autophagy was beneficial in primary RGCs and *C. elegans* (Figs. 2f and 4k), we investigated if autophagy inhibition could curtail blindness in *Opa1*^ΔRGC/ΔRGC^ mice. Therefore, we genetically inhibited autophagy by reducing *Atg7* levels in the same subset of Opa1-depleted RGCs. To this end, we generated *Opa1*^fl/fl^::*Atg7*^fl/+^::Grik4-Cre mice (*Opa1*^ΔRGC/ΔRGC^::*Atg7*^ΔRGC/+^). A further crossing to mtYFP^flstop/flstop^ mice allowed to isolate RGCs and confirm the expected reduction of *Atg7* mRNA levels (Supplementary Fig. 6c, d). Similar to what observed in primary *Atg7*^fl/+^ RGCs, autophagosomes were reduced and evenly distributed in cells with reduced OPA1 in retinas from 4-month-old *Opa1*^ΔRGC/ΔRGC^::*Atg7*^ΔRGC/+^ mice, (Fig. 5d–f). Furthermore, the increased levels of LC3B measured by flow cytometry in mtYFP^+^*Opa1*^ΔRGC/ΔRGC^ RGCs were also normalized in mtYFP^+^*Opa1*^ΔRGC/ΔRGC^::*Atg7*^ΔRGC/+^ cells (Fig. 5g). This autophagy inhibition was accompanied by a remarkable protection against the onset of visual defects. Unlike *Opa1*^ΔRGC/ΔRGC^ animals, *Opa1*^ΔRGC/ΔRGC^::*Atg7*^ΔRGC/+^ mice did not develop any visual defect at 4 months (Fig. 5a, c) and the protection was sustained for up to 12 months of age, at all spatial frequencies tested in the optokinetics assay (Supplementary Fig. 6f-m) and in the vision guided swimming test (Fig. 5b). In conclusion, *Atg7-*depletion curtails the visual loss caused by *Opa1* deletion in RGCs.

## Discussion

Here, we unveil a role for autophagy in the control of mitochondrial content in axons and in visual loss in a mouse model of ADOA caused by *Opa1* deletion. To which extent autophagy contributes to neurological diseases caused by primary mitochondrial dysfunction is unclear. While an increase in autophagy has been reported in other ADOA animal models, it was not clear whether it played any role in the pathogenesis of the disease^18,19^. Our data demonstrate that excess autophagy is detrimental in three different models of ADOA: primary mouse RGCs and *C. elegans* GABAergic neurons expressing pathogenic, dominant negative *Opa1* mutants, and in a mouse model of ADOA generated by RGC specific *Opa1* deletion. Autophagosomes accumulate and mitophagy is induced in fibroblasts derived from patients with the plus variant of ADOA^50^, caused by the dominant negative type of mutations used here. Our in vivo mouse data suggest that autophagy inhibition can be a therapeutic approach also for the more common and less severe forms of ADOA caused by *OPA1* haploinsufficiency. This possibility needs to be rigorously tested in vivo using models closer to human RGCs, e.g., iPSCs derived from patients^51,52^.

The concept that autophagy inhibition can be a therapeutic approach in ADOA is counterintuitive. Indeed, autophagy activation protects against accumulation of toxic protein aggregates that represent a cause and a hallmark of neurodegeneration. However, a primary mitochondrial defect like the one studied here could cause dysregulated autophagy ultimately curtailing the content of organelles and molecules in axons.

Mitochondrial trafficking in polarized cells like neurons is highly regulated^25^. Our results add mitophagy to the list of mechanisms controlling mitochondrial distribution at least in mouse RGCs and in different types of *C. elegans* neurons. Whether this model can be extended to other cell types, organelles, and diseases remains to be explored. However, clustering and abnormal distribution of mitochondria has been observed e.g. in Alzheimer’s disease models^53,54^. Moreover, RGCs are long-axon neurons like the superordinate centers neurons of the systems affected in Parkinson’s disease and the motoneurons affected in amyotrophic lateral sclerosis and Charcot–Marie–Tooth 2a^55^. In these diseases, trafficking of mitochondria is altered^26^ and autophagosomes aggregate in the soma^56,57^. Therefore, the role of autophagy in controlling axonal mitochondrial density could be investigated in other neurological disorders where mitochondria are defective and neurons with a long axon are affected.

Because *Atg7* depletion permanently protects from the visual loss caused by *Opa1* deletion, we conclude that mitochondria with reduced *Opa1* levels can nevertheless sustain RGC function throughout rodent life. RGCs constitute the innermost retinal cell layer and are hence directly exposed to the vitreous: intravitreal delivery of autophagy inhibitors, or of drugs modulating AMPK activity might offer a therapeutic strategy to curtail the visual loss in ADOA patients.

## Methods

### Plasmids and molecular biology

peYFP-hLC3 (YFP-LC3), pEYFP-Mito (mtYFP), mito-dsRED (mtRFP), pEGFP (GFP), pMSCV, pMSCV-Opa1, pMSCV-Opa1^K301A^ pMSCV-Opa1^Q297^, and pMSCV-Opa1^R905*^ were previously generated^29,32,58^. pMSCV-Cre-GFP and Cherry-Tubulin were obtained from Addgene. mt-mKEIMA was a kind gift from E. Ziviani (University of Padua, Italy). To generate pCMV-AMPK^T172A^, site directed mutagenesis was performed using the following primer sequence: 5′-GAATTTTTAAGAGCAAGTTGTGGCTC-3′ and 5′-GAGCCACAACTTGCTCTTAAAAATTC-3′.

YFP^+^ cells were isolated from mitoYFP^+^ mice using a FACSAria sorter (BD Bioscience) and mRNA was extracted using the miRNeasy Mini Kit (Qiagen). *Opa1* and *Atg7* levels were quantified by real-time amplification using the Power SYBR Green PCR Master Mix (Invitrogen) and normalized to actin levels. The following primers were used: for *Opa1*: 5′-ATACTGGGATCTGCTGTTGG-3′ and 5′-AAGTCAGGCACAATCCACTT-3′; for *Atg7*: 5′-ATGCCTTATGATGATCTGTGTTCG-3′ and 5′-ATCTTTGTCCTTTGACCTTGGA-3′; for actin: 5′-CTGGCTCCTAGCACCATGAAGAT-3′ and 5′-GGTGGACAGTGAGGCCAGGAT-3′.

To generate p mec-7DsRed::LGG-1 construct, we inserted an AgeI/EcoRI fragment (derived from plgg-1DsRed::LGG-142), containing the coding sequence of DsRed downstream of the mec-7 promoter of the pPD96.41 plasmid vector. We then fused an EcoRI fragment (derived from plgg-1DsRed::LGG-1) containing the coding sequence of lgg-1 at the carboxy terminus of DsRed of the pmec-7DsRed. To generate pmec-7mtGFP reporter construct, we inserted an EcoRI/BamHI fragment (derived from pPD96.32 plasmid vector), containing the coding sequence of GFP with an amino terminal mitochondrial localization signal downstream of mec-7 promoter of the pPD96.41 plasmid vector. The translational pmec-7DsRed::LGG-1 fusion construct was co-injected with pmec-7mtGFP and pRF4 (contains the rol-6(su1006) dominant transformation marker) into the gonads of wild-type animals. To generate the punc-47Opa1^K301A^ construct, we inserted an EcoRV fragment containing the Opa1^K301A^ sequence downstream of the unc-47 promoter in PCRII-TOPO vector. The final punc-47Opa1^K301A^ construct was co-injected with pRF4 (contains the rol-6(su1006) dominant transformation marker) into the gonads of wild-type animals. To generate the punc-119TOMM-20::Rosella reporter construct, we removed the myo-3 promoter from pmyo-3TOMM-20::Rosella plasmid and inserted a HindIII/XbaI fragment containing the sequence of unc-119 promoter (derived from punc-119CTS-1::GFP). The translational punc-119TOMM-20::Rosella fusion construct was co-injected with pRF4 into the gonads of wild-type animals.

### *C. elegans* strains and genetics

We followed standard procedures for *C. elegans* strain maintenance. Nematode rearing temperature was kept at 20 °C. The following strains were used in this study: N2: wild-type Bristol isolate, RB754: aak-2(ok524)X, lgg-2(tm5755)IV. To monitor mitochondrial number and distribution in neurons, we used IR1505: N2; Ex001[punc-119CTS-1::mCherry]^59^, IR1766: lgg2-(tm5755)IV; Ex001[punc-119CTS-1::mCherry], IR1493: N2; Ex001[punc-119CTS-1::GFP], IR2105: lgg2-(tm5755)IV; Ex001[punc-119CTS-1::GFP], IR1776: N2; Ex001[pmec-17mtGFP; pmec-17mCherry], IR1803: lgg-2(tm5755)IV; Ex001[pmec-17mtGFP; pmec-17mCherry], EG6531: N2; oxIs608[punc-47mCherry]; oxEx1182[punc-47TOMM-20::GFP]69, IR1774: lgg-2(tm5755)IV; oxIs608[punc-47mCherry]; oxEx1182[punc-47TOMM-20::GFP], IR1851: aak-2(ok524)X; oxIs608[punc-47mCherry]; oxEx1182[punc-47TOMM-20::GFP], IR1826: dct-1(tm376)X; oxIs608[punc-47mCherry]; oxEx1182[punc-47TOMM-20::GFP, IR2207: isp-1(qm150)IV; oxIs608[punc-47mCherry]; oxEx1182[punc-47TOMM-20::GFP], IR2097: N2; oxIs608[punc-47mCherry]; oxEx1182[punc-47TOMM-20::GFP]; Ex0023[punc-47Opa1^K301A^] and IR2110: lgg-2(tm5755)IV; oxIs608[punc-47mCherry]; oxEx1182[punc-47TOMM-20::GFP]; Ex0023[punc-47Opa1^K301A^] strains. To monitor autophagy, we used VIG9: unc119(ed3)III; Is[unc-119(+); plgg-2GFP::LGG-2] and DA2123: N2; adIs2122[plgg-1GFP::LGG-1; rol-6(df)]II strains. The following strains were used to monitor the neuronal mitophagy process: IR1797: N2;Ex001[pmec-7mtGFP; p mec-7DsRed::LGG-1], and IR1864: N2; Ex001[punc-119TOMM-20::Rosella] strains. To investigate the defecation motor program, we used IR2093: N2; Ex0023[punc-47Opa1^K301A^] and lgg-2(tm5755)IV; Ex0023[punc-47Opa1^K301A^] strains.

### Generation of mouse models and animal handling

Opa1^fl/fl^ mice^32^ were crossed with Grik4-Cre mice (Jackson laboratories) and with Atg7^fl/fl^ mice (M. Komatsu, Tokyo Metropolitan Institute of Medical Science, Japan)^39^. For flow cytometry experiments, a Cre-inducible mito-YFP mouse, Gt(ROSA)26Sor+/lox-Stoplox-mito-YFP^35^, was crossed with Opa1^fl/fl^, Atg7^fl/fl^ and Grik4-Cre mice. 3- to 12-month-old mice were used to test visual acuity. Genotypes were assessed using tail genomic DNA and the following primers. For Opa1^fl/fl^: 5′-CAGTGT TGATGACAGCTCAG-3′; and 5′-CATCACACACTAGCT TACATTTGC-3′; for Cre: 5′-GCGGTCTGGCAGTAAAAACTA TC-3′; and 5′-GTGAAACAGCATTGCTGTCACTT-3′; for Atg7^fl/fl^: 5′-TGGCTGCTACTTCTGCAATGATGT-3′; and 5′-CAGGACAGAGACCATCAGCTCCAC-3′; for YFP: 5′-AAAGTCGCTCTGAGTTGTTAT-3′; and 5′-GCGAAGAGTTTGTCCTCAACC-3′; 5′-GGAGCGGGAGAAATGGATATG-3′. All mouse procedures were performed according to approved protocols (protocol 32/2011 CEASA University of Padua and 318/2015 Italian Ministry of Health to L.S.). Mice (maximum 4/cage, with environmental enrichment) were housed in a conventional mouse facility with 12 h light/dark cycles at a constant temperature of 22 °C and controlled humidity varying between 30 and 50%. Mice were checked daily by the Veneto Institute of Molecular Medicine veterinarian for well-being.

### RGC purification and culture

Retinas from 8–10 P0–P2 C57Bl/6J were dissected and dissociated incubating with 15 U/ml papain (Worthington) for 30 min. RGC were purified via immunopanning-magnetic separation using macrophage antiserum (Fitzgeral Industries) and anti-Thy1 antibody (MiltenyiBiotec)^31^. Totally, 10^4^ cells were transfected with the indicated plasmids using Neon Transfection System (Invitrogen), and seeded onto 13- or 24-mm round glass coverslips coated with poly-l-ornithine 0.2 mg/ml (Sigma) and laminin 0.5 mg/ml (Roche). Cells were maintained in Neurobasal A Medium (Invitrogen) containing B-27 (Gibco), N-2 (Invitrogen), 1% l-glutamine (Invitrogen) and 25 ng/ml nerve growth factor (BD Bioscience) at 37 °C in a 5% CO_2_ atmosphere. Autophagy was inhibited with 200 nM Bafilomycin A (Sigma) for 30 min or induced with 100 nM Rapamycin (Sigma) for 30 min.

### Imaging

For confocal imaging, 10^4^ cells seeded onto 24-mm round glass coverslips were transfected with the indicated plasmids. After 24 h, cells were placed on the stage of a laser scanning microscope (TCS SP5, Leica). Using the LasAF software (Leica), RFP and YFP were excited using the 488 nm or the 543 nm line of the HeNe and Argon with a 63×, 1.4NA objective (Leica). Confocal images of mtRFP and YFP fluorescence were acquired along the *z*-axis, deconvolved and 3D reconstructed using the Convolve and the VolumeJ plugins of ImageJ (National Institutes of Health, Bethesda). For confocal imaging of retinal sections, tetramethylrhodamine (TRITC), fluoresceinisothiocyanate (FITC), and 4′,6-diamidin-2-phenylindole (DAPI) were excited using the 488 nm, 543 nm, or 405 line of the HeNe, Argon, and Diode 405 with a 63×, 1.4NA objective. Confocal images were acquired along the *z*-axis and z-projected using Image J. Colocalization between autophagosomes and mitochondria was quantified using Manders’ coefficient^10^ on individual z-planes of stacks of confocal images. Length of mitochondria and axons were manually traced. Length and number of mitochondria were quantified using the Multimeasure plug-in of Image J. All mitochondria retrieved in entire axon length were considered in the analysis. The distribution of autophagosomes and mitochondria was measured on manually traced somas in primary RGCs and retinal sections using the “skewness” analysis of ImageJ. *Opa1*^ΔRGC/ΔRGC^ RGCs were identified by reduced staining of OPA1 in ADOA mice while all cells of the ganglion cell layer were analyzed in WT mice.

For mitophagy measurements, RGCs were cotransfected with mt-mKEIMA^34^ and the indicated plasmids. Samples were imaged using a laser scanning microscope (TCS SP5, Leica) equipped with a 40×, 1.25NA objective (Leica). mt-mKEIMA was excited by using the 438 nm or the 550 nm line of the HeNe and Argon laser and the emission was collected at 600 nm. Confocal planes were acquired along the *z*-axis and automatically segmented by the “segmentation” plugin of ImageJ. The 550/438 ratio was used to measure mitophagy.

For mitochondrial imaging in *C. elegans*, 4-day-old adult hermaphrodites co-expressing in GABA motor neurons mtGFP and cytoplasmic mCherry were exposed to 10 mM paraquat and imaged after 2 days at 20 °C. Worms were immobilized with levamisole before mounting on 2% agarose pads for microscopic examination with a Zeiss AxioObserver Z1 confocal microscope. Imaging parameters such as microscope and camera settings (lens and magnifier used, filters exposure time, resolution, laser intensity, gain, etc.) were kept constant for all the animals imaged. Quantification of mitochondrial number was performed using ZeissZEN 2012 software to automatically threshold the images and determine the outlines of GFP-targeted mitochondria in axons of GABA motor neurons. Mitochondrial number was calculated by counting the average number of puncta per 100 μm of axonal length.

### Immunofluorescence

Primary RGCs were seeded onto 13-mm round, poly-l-ornithine and laminin coated glass coverslips. After 24 h, cells were treated as indicated and fixed for 10 min at room temperature with 3.7% (w/v) formaldehyde (Sigma). For retinal slices, mice were sacrificed, eyes dissected, and fixed O/N at 4 °C with 3.7% (w/v) formaldehyde. Retinas were cryoprotected in a sucrose gradient (10, 20, and 30% w/v in PBS, Sigma) and 10 μm thick cryostat sections were cut. Cells and sections were permeabilized for 10 min with 0.1% Triton-X-100 (Sigma), blocked for 1 h with BSA 1% and incubated with primary antibodies. Staining was revealed with a goat anti-rabbit or anti-mouse IgG conjugated to FITC or TRITC.

The following antibodies were used: Atg7 (Sigma A2856, 1:500), Brn3a (Santa Cruz Biotechnology sc-31984, 1:500), GFP (Invitrogen A11120 and A11122, 1:1000), LC3B (Nanotools 0231, 1:500; Cell Signalling 2775 1:500), Phospho-AMPKα (Thr172) (Cell Signalling 2535, 1:200), β-Tubulin III (Sigma T8660, 1:1000), Ulk1 (Novus Biologicals, NBP2-41217, 1:200). Opa1 antibody^11^ was from Dr. A. van der Bliek (University of California, Los Angeles, USA, 1:200).

### Apoptosis

RGCs were seeded onto 13-mm round glass coverslips coated with poly-l-ornithine and laminin. After 24 h cells were fixed for 10 min at room temperature with 3.7% (w/V) formaldehyde and permeabilized for 10 min with Triton-X-100 0.1%. Apoptosis was evaluated by TUNEL using the In-Situ Cell Death Detection Kit (Roche) in FITC or TRITC.

### Flow cytometry

mtYFP^+^, Opa1^fl/fl^::mtYFP^+^::Cre^+/−^, Opa1^fl/fl^::Cre^+/−^::mtYFP^+^ and Opa1^fl/fl^::Atg7^fl/+^::Cre^+/−^::mtYFP^+^ mice were euthanized. Retinas were dissected and dissociated with 15 U/ml papain (Worthington) at 33 °C for 30 min. Then, cells were fixed with 1% paraformaldehyde (Sigma) for 30 min at 4 °C, blocked and permeabilized with 5% BSA and 1% Triton-X-100 for 1 h at 4 °C. Primary antibodies were incubated in 2.5% BSA and 0.01% Triton-X-100 for 1 h at 4 °C and secondary antibodies in 5% BSA and 0.05% Triton-X-100 for 30 min at 4 °C. The following primary antibodies were used: Bnr3a (Abcam ab81213, 1:200) and GFP (Invitrogen A11122, 1:100). The following secondary antibodies were used: goat anti-mouse Alexa Fluor 405 (Invitrogen A-31553, 1:700) and goat anti-rabbit Alexa Fluor 594 (Invitrogen A-11005, 1:500). Cells were gated using FSC-A vs SSC-A by size and granularity (BD FACS Canto II). YFP^+^ and Brn3a^+^ cells were identified by excluding the positivity of cells marked solely with secondary antibodies. Data were analyzed using BD FACS Diva Software. Brn3a^+^YFP-405^+^ cells were ~600.

### Optokinetic test

Visual acuity was measured using an Argos Optokinetic Drum (Instead technologies). The mice were placed on a platform surrounded by 4 monitors in a dark chamber provided with a silent ventilation system. The monitors were controlled by a computer, which automatically selected the type of stimulus. The stimulus consisted on alternated black and white vertical stripes which varied in size and velocity of presentation. The size was represented by the thickness of stripes ranging from 0.01 to 0.7 A.U. and the frequency of rotation of the stripes ranged from 0.1 to 6.4 Hz. The program randomly presented the stripes clockwise or counterclockwise and mice were recorded by a camera. The experimenter decided if mice performed an optomotor movement or not and in which direction. The system was blind because the experimenter did not see the stimuli. The computer compared the answers of the experimenter to the stimulus presented and if they matched, the counter of successful answers increased. 10 stimuli were presented for each condition and at the end of the presentation the computer displayed the percentage of correct visual responses. Mice were considered blind when their correct responses were <80%. The optokinetic reflex did not occur in wild-type animals to the frequency of 6.4 Hz and the distance of 0.7 A.U. These parameters were used as negative control.

### Vision-guided forced swimming test

Two monitors were placed side-by-side at the wide end of a Y-shaped water tank. Screens brightness was calibrated and adjusted so that they were equal. Vertical stripes were displayed on one of the monitors with a pseudo-random pattern, such as LRLLRLRR (L—left monitor, R—right monitor), while the other displayed homogeneous gray of the same mean luminance. The hidden platform was always placed directly below the monitor displaying the stripes. Mice were trained to swim towards the monitors and associate the striping with a hidden platform that allows them to escape from water. Ability to find the platform indicates capability to resolve the visual task. The end of the midline divider that separates the stimuli was considered the decision point. Mice were released from the release point placed at the short end of the pool and swam towards the monitors. Once the decision point had been crossed, the choice was considered drawn and scored (correct or error). Mice were considered to have learned the task when they achieved a pass rate of 80%. The experimenter was blinded to the genotype of the tested mice.

### Statistical analysis

The sample size was not predetermined using statistical methods. No animals were excluded from the analysis. The experiments were not randomized. The investigators were blinded to sample identity and outcome assessment. N of independent experiments and *n* of cells examined for each condition are indicated. In box plots, centre line represents mean, bounds of boxes SEM, whiskers the 10th–90th percentiles; each dot represents an individual nematode form the indicated *N* of independent experiments otherwise indicated. Prism 8.3 (GraphPad Software) or Origin 2019b (OriginLab) were used for statistical analyses. In other graphs, average ± SEM is plotted. Statistical significance was calculated by one-way or two-way ANOVA test with Bonferroni mean comparison between the indicated samples. Nonparametric ANOVA test were applied if assumptions of normality and homoscedasticity were not respected. *p* Values are indicated in the legends. A *p* value < 0.05 was considered statistically significant.

### Reporting summary

Further information on research design is available in the Nature Research Reporting Summary linked to this article.

## Supplementary information

Supplementary Information

Peer Review File

Movie 1

Movie 2

Movie 3

Reporting Summary

## Data Availability

The Source Data underlying the following Figs. 1b, c, e, f; 2b, d, e, g; 3b, c, e, f, g; 4b, d, f, h, j, k; 5a, c, e, f, g; and Supplementary Figs. 1b, d, f; 2b, d, f; 3b; 4b, c, d; 5b, d, e–m are provided in the Source Data File. All data are available from the corresponding author upon reasonable request. Source data are provided with this paper. Source data are provided with this paper.

## Source data

Source Data
