# Supplementary material for: Inhibition of autophagy curtails visual loss in a model of autosomal dominant optic atrophy
Source: Nat Commun. 2020 Aug 12;11:4029. doi: 10.1038/s41467-020-17821-1 (PMC7423926; doi:10.1038/s41467-020-17821-1)
Supplement: Supplementary file 1 — Supplementary Information [file 41467_2020_17821_MOESM1_ESM.pdf]

SUPPLEMENTARY INFORMATION

Inhibition of autophagy curtails visual loss in a model of autosomal dominant optic atrophy

Zaninello et al.

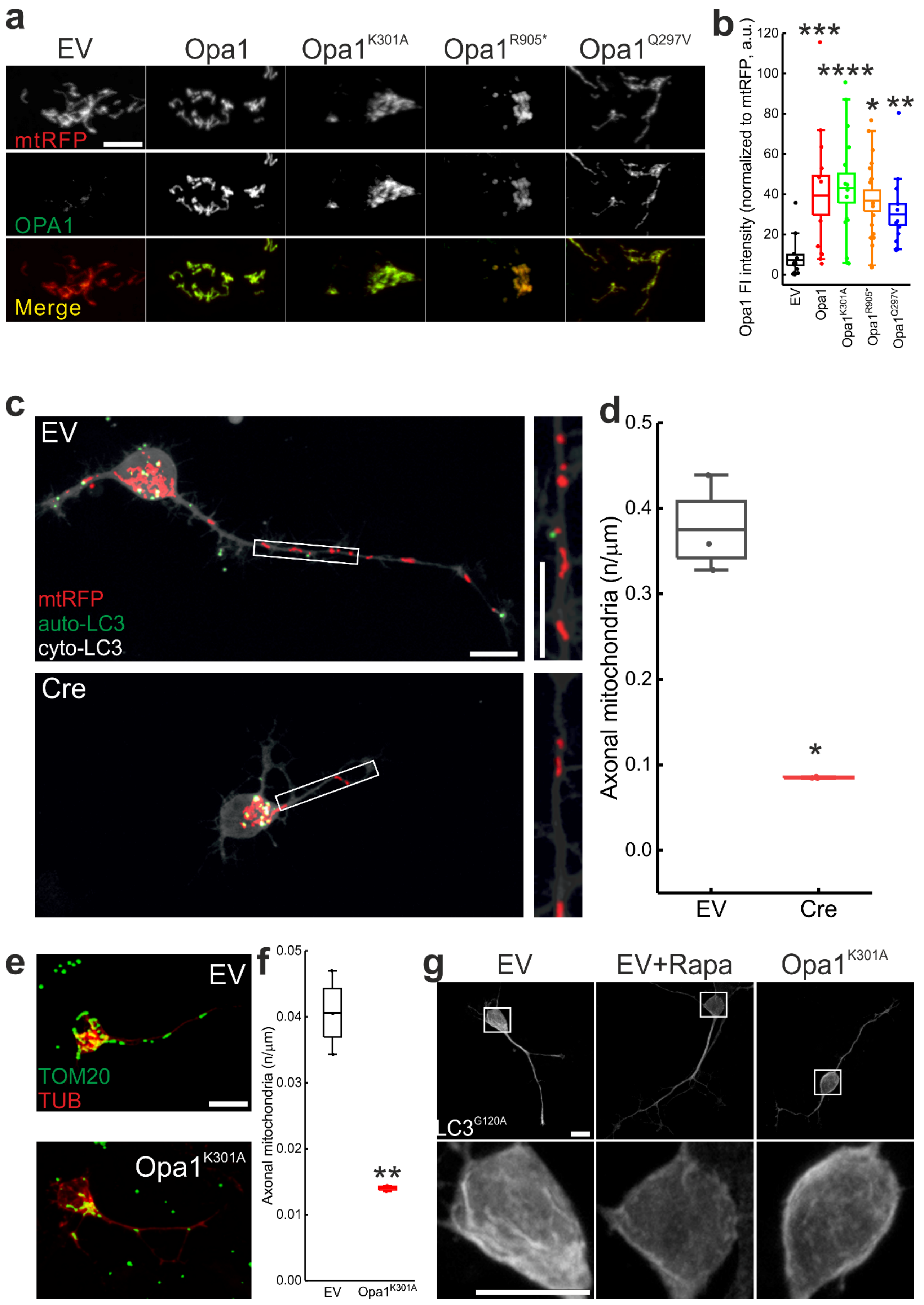

Supplementary Figure 1. Characterization of primary mouse RGCs as ADOA models, related to Fig. 1.

(a) Representative z-projections of stacks of confocal images of the fluorescence of mtRFP (red) in primary RGCs co-transfected with the indicated plasmids and stained for OPA1 (green). EV, empty vector. Bar, 20 µm.
(b) OPA1 fluorescence intensity (FI) in the soma of mtRFP^{+} RGCs from 4 independent experiments as in (a). EV, n=14; Opa1, n=13; Opa1^{K301A}, n=16; Opa1^{R905*}, n=17; Opa1^{Q927V}, n=12 cells. *, p=0.016; **, p=0.04; ***, p=0.004; ****, p=0.0002 vs EV in a one-way ANOVA test followed by Kruskal-Wallis post hoc test.
(c) Representative z-projections of stacks of confocal images of the fluorescence of mtRFP (red) and YFP-LC3 (green, autophagosome-LC3, auto-LC3) in primary *Opa1*$^{fl/fl}$ RGCs cotransfected the indicated plasmids. The cytoplasmic YFP-LC3 signal (cyto-LC3) is pseudocolored in grey for the sake of clarity. The boxed area is magnified on the right to highlight mitochondrial density in axons. Bars, 20 µm.
(d) Quantification of mitochondrial axonal density from 3 independent experiments as in (c). EV, n=60; Cre, n=60 cells. *, p=0.013 vs. EV in a t-test followed by Welch's post hoc test.
(e) Representative z-projections of stacks of confocal images of Cherry-Tubulin (red) in primary RGCs co-transfected with the indicated plasmids and after 24 h fixed and immunostained with TOM20 (green). Bar, 20 µm.
(f) Quantification of mitochondrial axonal density in 3 independent experiments as in (e). EV, n=64; Opa1^{K301A}, n=58 cells. **, p=0.009 vs EV in a t-test followed by Welch's post hoc test. In Box plots, centre line represents mean, bounds of boxes SEM, whiskers the 10th-90th percentiles; each dot represents independent experiments. Source data are provided as a Source Data file.
(g) Representative z-projections of stacks of confocal images of the fluorescence of GFP-LC3^{G120A} in primary RGCs cotransfected with the indicated plasmids, from N=3 independent experiments. Where indicated, cells were treated with Rapamycin (Rapa, 100 nM) for 30 min to activate autophagy. Somas were enlarged in the bottom panels. Bar, 20 µm.

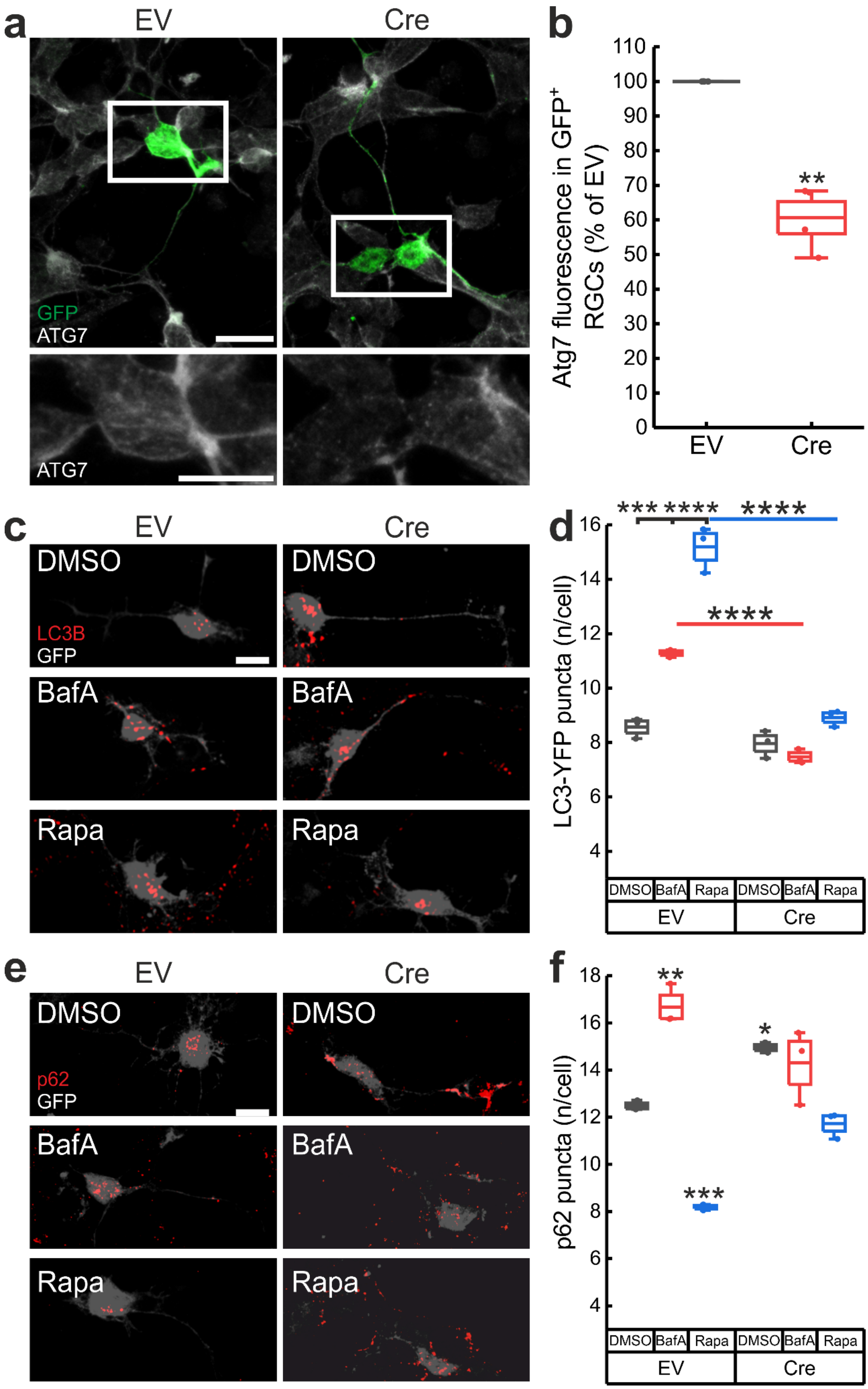

Supplementary Figure 2. Decreased autophagic flux in primary *Atg7*$^{fl/+}$ RGCs, related to Fig. 2.

(a) Representative z-projections of stacks of confocal images of the fluorescence of ATG7 (grey) and GFP (green) in primary *Atg7*$^{fl/+}$ RGCs cotransfected with GFP and the indicated plasmids. Bottom images: soma. EV, empty vector. Bars, 20 μm.
(b) Quantification of ATG7 fluorescence levels from 4 independent experiments as in (a). EV, n=99; Cre, n=101cells. **, p=0.0034 vs EV in a t- test followed by Welch's post hoc test.
(c,e) Representative z-projections of stacks of confocal images of the fluorescence of GFP (grey) of primary Atg7$^{fl/+}$ RGCs co-transfected with the indicated plasmids and after 24 hrs treated as indicated, fixed and immunostained for LC3B (c) or p62 (e) (red). BafA, bafilomycin A; Rapa, rapamycin. Bars, 20 μm.
(d) Autophagic flux from 3 independent experiments as in (c). For EV: DMSO, n=67; BafA, n=53; Rapa, n=81 cells; for Cre: DMSO, n=82; BafA, n=54; Rapa, n=75 cells; ***, p=0.0002; ****, p<0.0001 vs. the indicated group in a two-way ANOVA test followed by Sidak's post hoc test
(f) Autophagic flux from 3 independent experiments as in (e). For EV: DMSO, n=63; BafA, n=60; Rapa, n=71 cells; for Cre: DMSO, n=61; BafA, n=63; Rapa, n=78 cells. *, p=0.021; **, p=0.0003; ***, p=0.0002 vs. (EV, DMSO) in a two-way ANOVA followed by Tukey's post hoc test. In Box plots, centre line represents mean, bounds of boxes SEM, whiskers the 10th-90th percentiles; each dot represents independent experiments. Source data are provided as a Source Data file.

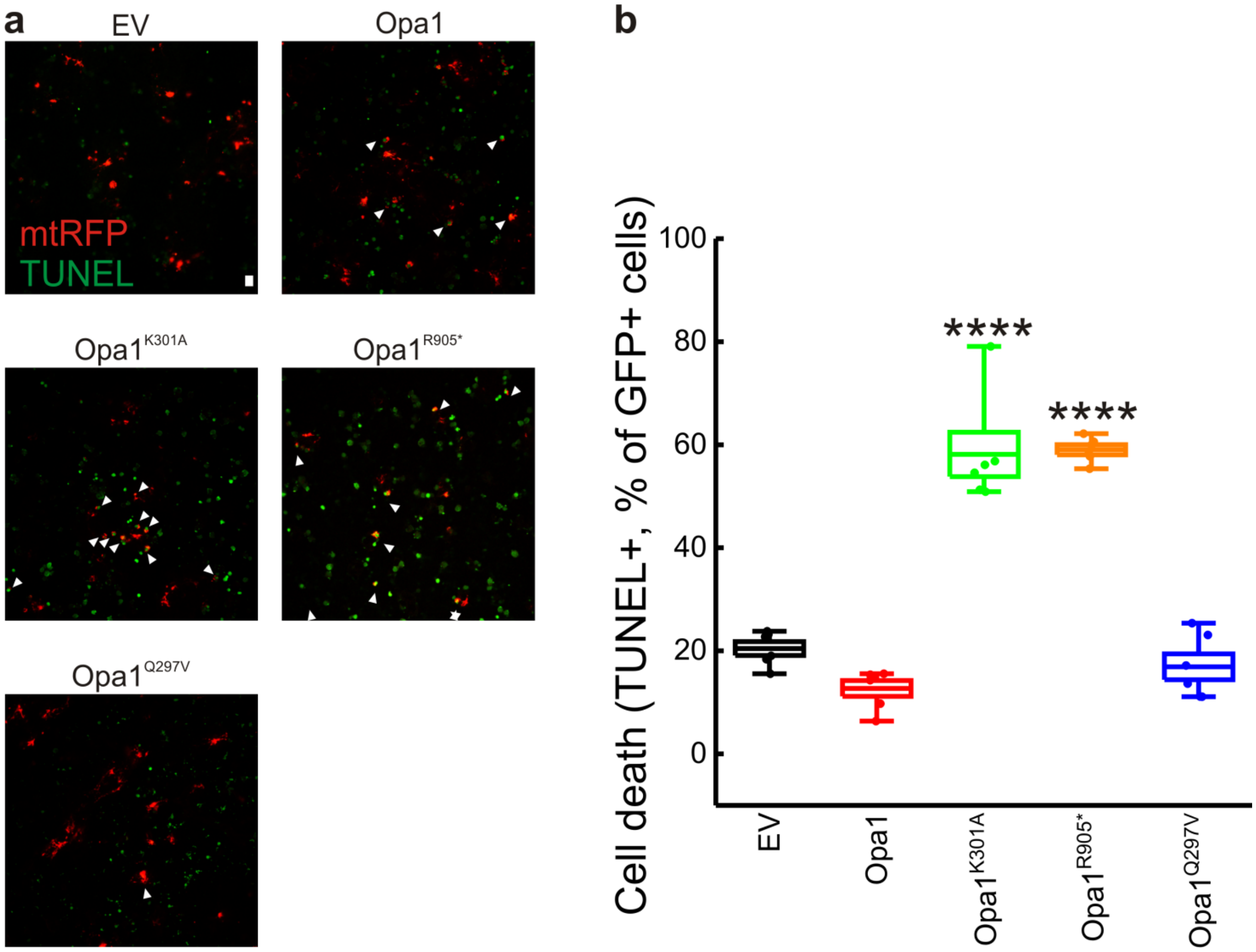

Supplementary Figure 3. Pathogenic mutants induce apoptosis of primary RGCs, related to Fig. 2.

(a) Representative confocal images of RGCs overexpressing mtRFP (red) and the indicated plasmids stained for apoptotic nuclei (TUNEL, green) 24 hrs after transfection. White arrows indicate apoptotic RGCs. EV, empty vector. Bar, 20 µm.

(b) Quantification of apoptosis from 5 independent experiments (n=40 cells/experiment) as in (a). ****, $p<0.0001$ in a parametric two-way ANOVA test followed by Tukey's post hoc test. In Box plots, centre line represents mean, bounds of boxes SEM, whiskers the 10th-90th percentiles; each dot represents independent experiments. Source data are provided as a Source Data file.

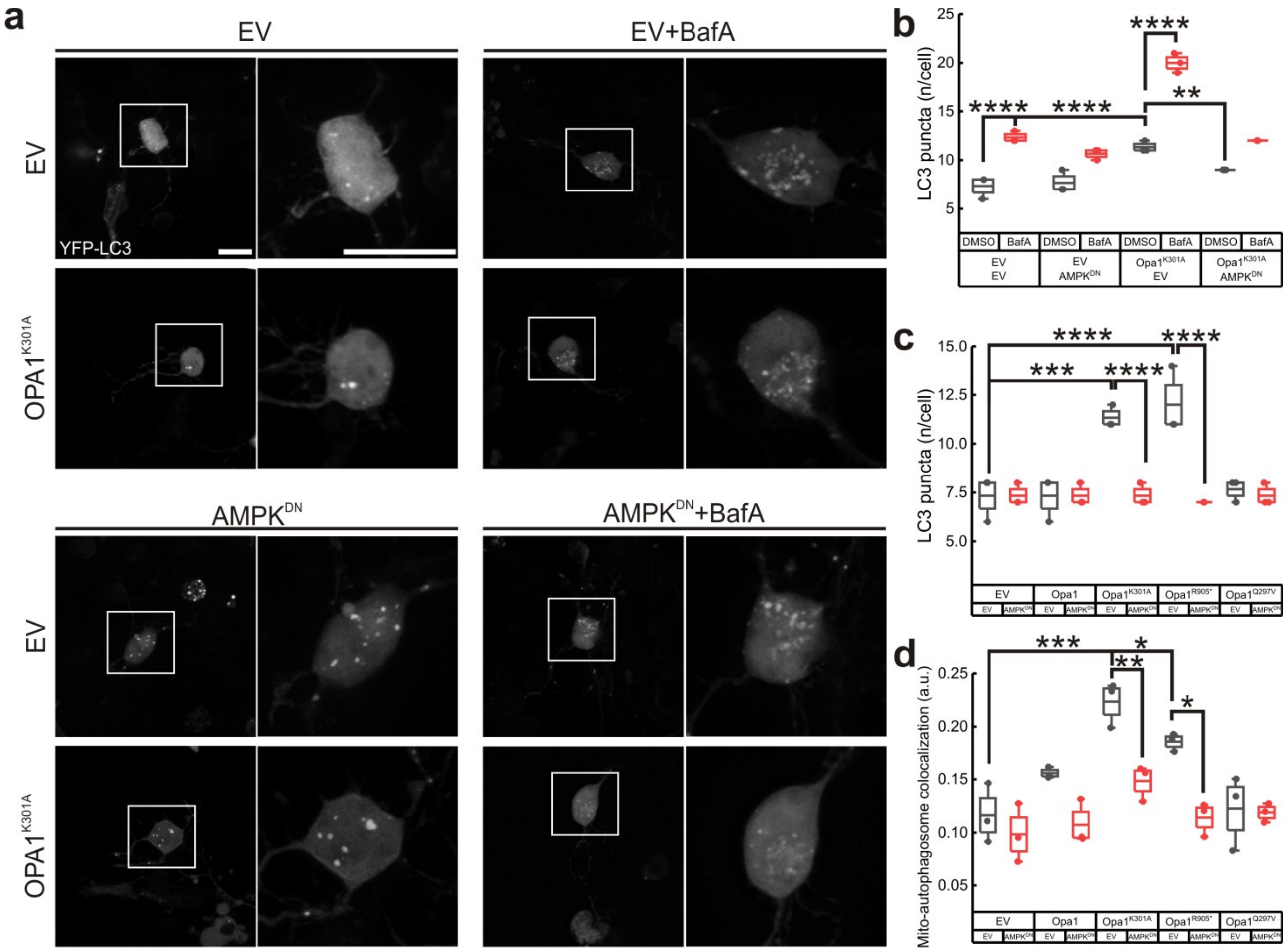

Supplementary Figure 4. AMPK^{DN} decreases autophagic flux in Opa1^{K301A} expressing RGCs. Related to Fig. 3.

(a) Representative z-projections of stacks of confocal images of the fluorescence of YFP-LC3 (grey) in primary RGCs co-transfected with the indicated plasmids and treated with bafilomycin A (BafA). The right images depict the somas magnified 3 times. EV, empty vector. Bars, 20 µm.

(b) Quantification of autophagic flux from 3 independent experiments as in (a) Number of cells analyzed per conditions were: EV EV DMSO, 60; EV EV BafA, 55; EV AMPK^{DN} DMSO, 48; EV AMPK^{DN} BafA, 50; Opa1^{K301A} EV DMSO, 51; Opa1^{K301A} EV BafA, 52; Opa1^{K301A} AMPK^{DN} DMSO, 52; Opa1^{K301A} AMPK^{DN} BafA, 51. **, p=0.004; ***, p<0.0001 between the indicated samples in a two-way ANOVA test followed by Tukey's post hoc test.

(c) Quantification of autophagosomes in RGCs from 3 independent experiments as in Fig. 3d Number of cells analyzed per conditions were: EV EV, 56; EV AMPK^{DN}, 46; Opa1 EV, 53; Opa1 AMPK^{DN}, 47; Opa1^{K301A} EV, 52; Opa1^{K301A} AMPK^{DN} 60; Opa1^{R905*} EV, 50; Opa1^{R905*} AMPK^{DN}, 57; Opa1^{Q297V} EV, 51; Opa1^{Q297V} AMPK^{DN}, 52. ***, p= 0.0001; ****, p<0.0001 between the indicated samples in a two-way ANOVA test followed by Tukey's post hoc test.

(d) Quantification of autophagosome-mitochondria colocalization from 3 independent experiments as in Fig.3d. Number of cells analyzed per conditions were: EV EV, 51; EV AMPK^{DN}, 47; Opa1 EV, 43; Opa1 AMPK^{DN}, 47; Opa1^{K301A} EV, 58; Opa1^{K301A} AMPK^{DN} 59; Opa1^{R905*} EV, 38; Opa1^{R905*} EV vs. AMPK^{DN}, 51; Opa1^{Q297V} EV, 53; Opa1^{Q297V} AMPK^{DN}, 45. *, p=0.017 (Opa1^{R905*} vs. Opa1^{K301A}); *, p=0.013 (Opa1^{R905*}, EV vs. AMPK^{DN}); **, p=0.008; ***, p=0.0001 between the indicated samples in a two-way ANOVA test followed by Tukey's post hoc test. In Box plots, centre line represents mean, bounds of boxes SEM, whiskers the 10th-90th percentiles; each dot represents independent experiments. Source data are provided as Source Data file.

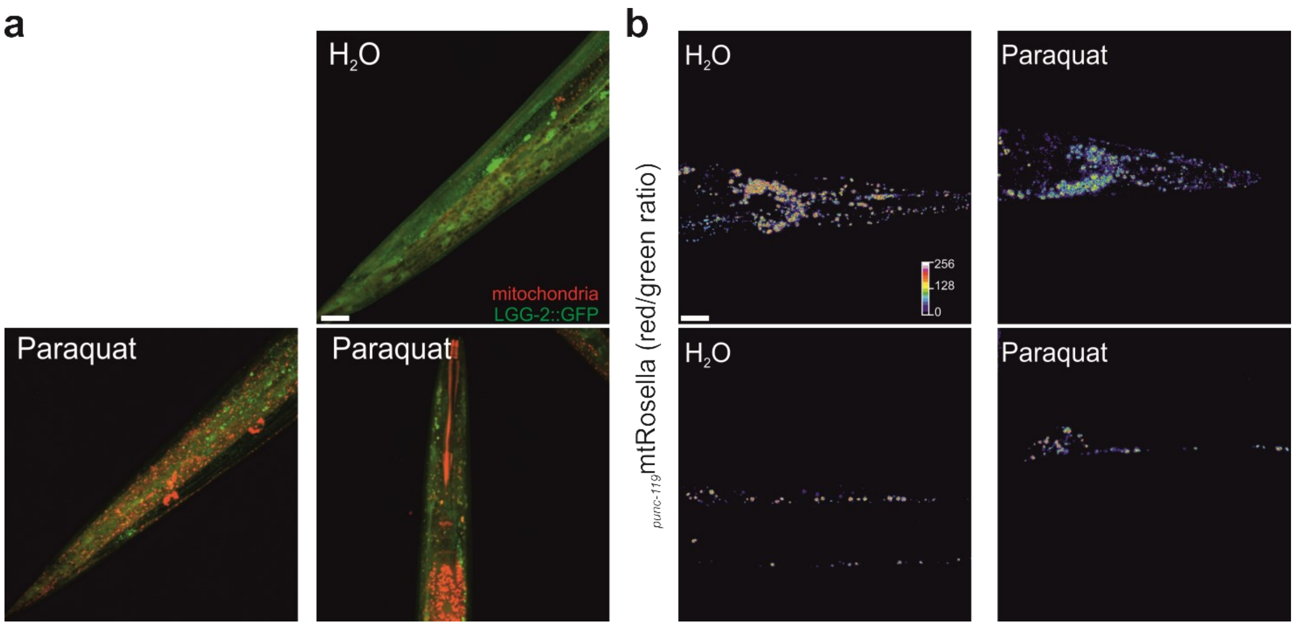

Supplementary figure 5. Autophagy controls mitochondrial content in C. elegans. Related to Fig. 4.

(a) Representative confocal images of the fluorescence of LGG-2 (green) of 3 independent experiments in animals exposed to paraquat (n=30 animals/genotype/condition). Animals were stained with TMRE to image mitochondria (red). Bar, 20 μm.

(b) Representative confocal images of the fluorescence of mitochondria-targeted Rosella biosensor in 3 independent experiments in animals treated with paraquat (n=30 animals/genotype/condition). Mitophagy stimulation is signified by the reduction of the ratio between pH-sensitive GFP to pH-insensitive DsRed. Bar, 20 μm.

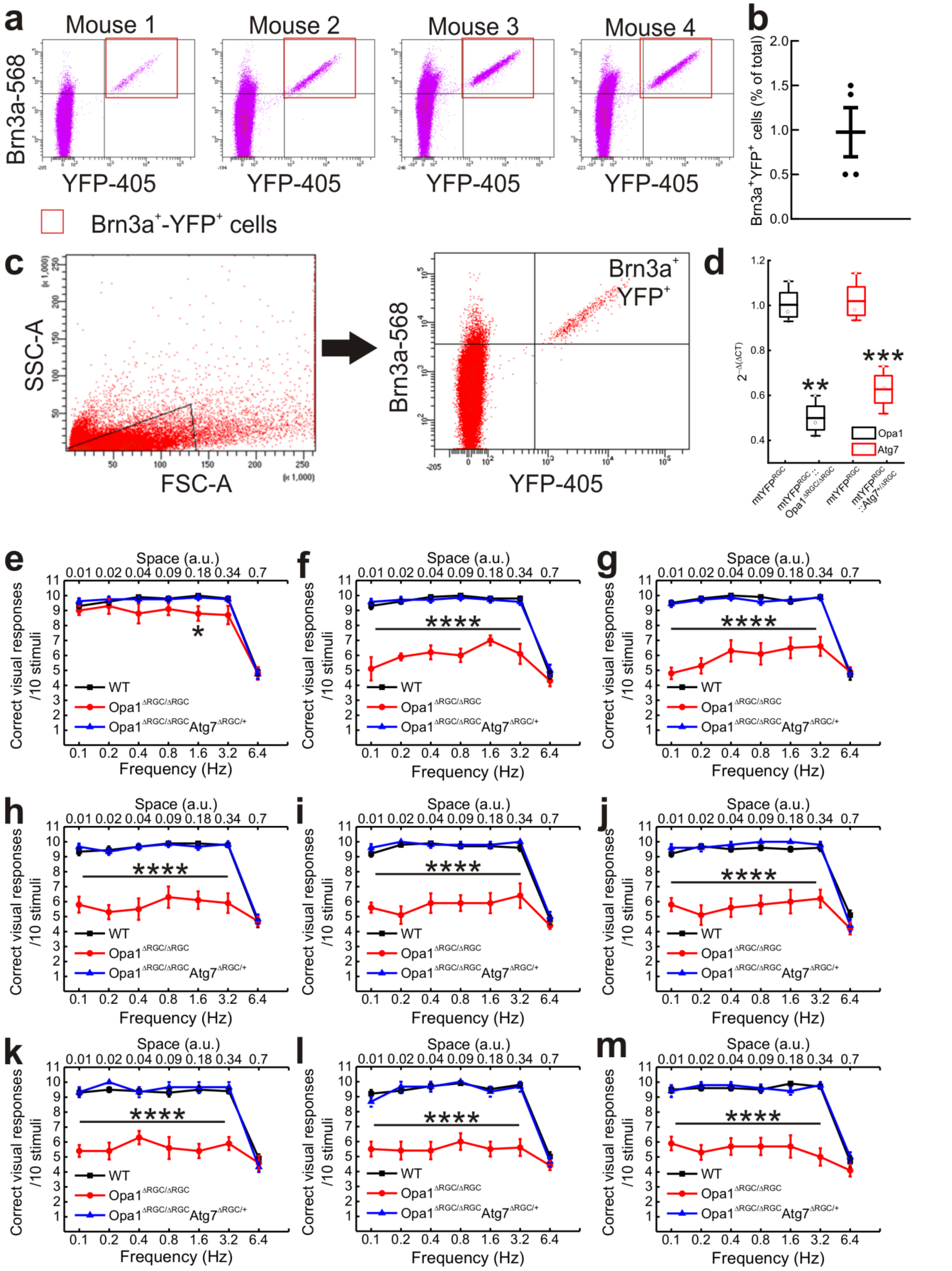

Supplementary figure 6. Characterization of *Opa1*$^{\Delta RGC/\Delta RGC}$ and *Opa1*$^{\Delta RGC/\Delta RGC}$::*Atg7*$^{\Delta RGC/+}$ mice. Related to Fig. 5.

(a) Whole retina cellular preparations from 4 different RGC $mtYFP^{+/+}$ mice (genotype: $mtYFP^{flstop/flstop}$::Grik4-$Cre^{+/-}$) were immunostained for the specific RGC marker Brn3a and analyzed by flow cytometry for mtYFP and Brn3a fluorescence.
(b) Percentage of double $mtYFP^{+}$, $Brn3a^{+}$ cells in experiments as in (a). Data represent average±SEM of 4 animals. Dots represent individual animals.
(**c**) A whole retina cellular preparation from a $Opa1^{\Delta RGC/\Delta RGC}Atg7^{\Delta RGC/+}mtYFP^{RGC/RGC}$ mouse (genotype: $Opa1^{flx/flx}Atg7^{flx/+}mtYFP^{flstop/flstop}$::Grik4-$Cre^{+/-}$) was immunostained for the specific RGC marker Brn3a and used to purify the $Brn3a^{+}$, $mtYFP^{+}$ cellular population by FACS. SSC-a, side scatter area; FSC-a, forward scatter area.
(d) mRNA levels of *Opa1* and *Atg7* determined by real time PCR (3 animals for Opa1 and 4 for Atg7) on RGCs sorted from $Opa1^{\Delta RGC/\Delta RGC}Atg7^{\Delta RGC/+}mtYFP^{RGC/RGC}$ mice as in (c). **, p=0.0013; ***, p=0.001 in a t-test wtih Welch's post hoc test. In Box plots, centre line represents mean, bounds of boxes SEM, whiskers the 10th-90th percentiles; each dot represents independent experiments.
(e-m) Average±SEM visual acuity in 3 (e) and 5-12 (f-m) months-old mice of the indicated genotype in the optokinetic test. Mice were subjected to 10 visual stimuli at the indicated temporal and spatial frequencies. Visual acuity is proportional to the n of correct answers to the stimulus. Number of mice tested was: WT, n=10 in all panels; $Opa1^{\Delta RGC/\Delta RGC}$ n=10 in (e-l), n=9 in (m); $Opa1^{\Delta RGC/\Delta RGC}Atg7^{\Delta RGC/+}$ n=8 (e, h, l); n=7 (f, g, i, j, k, l, m). *, p=0.029; ****, p<0.001 vs the corresponding WT frequency in a nonparametric one-way ANOVA test followed by Tukey's post hoc test. a.u., arbitrary units.
