# Supplementary figures and images for: Inhibition of autophagy curtails visual loss in a model of autosomal dominant optic atrophy
Source: Nat Commun. 2020 Aug 12;11:4029. doi: 10.1038/s41467-020-17821-1 (PMC7423926; doi:10.1038/s41467-020-17821-1)

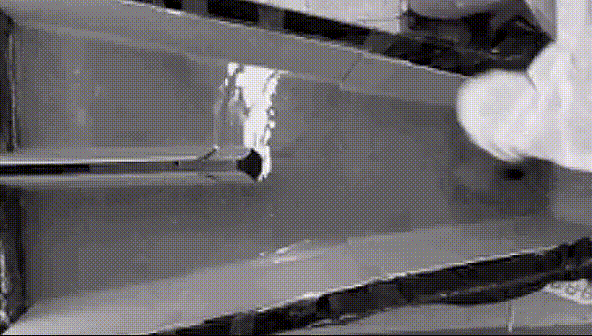

Supplement: Supplementary file 3 — Movie 1 [file 41467_2020_17821_MOESM3_ESM.gif]

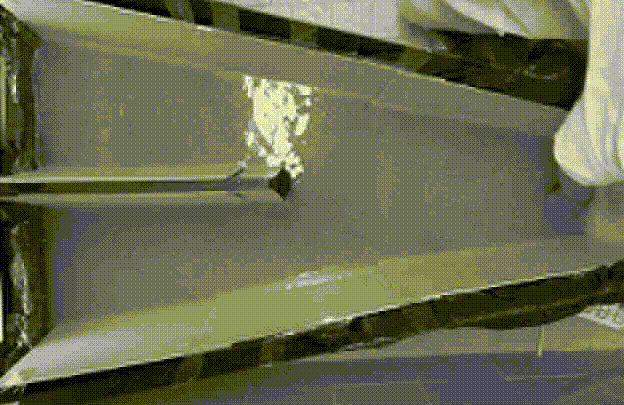

Supplement: Supplementary file 4 — Movie 2 [file 41467_2020_17821_MOESM4_ESM.gif]

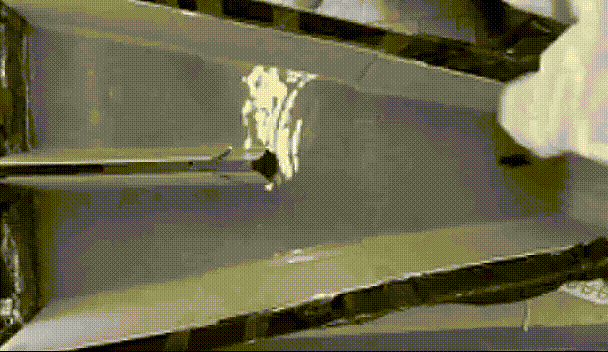

Supplement: Supplementary file 5 — Movie 3 [file 41467_2020_17821_MOESM5_ESM.gif]
