# Supplementary material for: Inhibition of autophagy curtails visual loss in a model of autosomal dominant optic atrophy
Source: Nat Commun. 2020 Aug 12;11:4029. doi: 10.1038/s41467-020-17821-1 (PMC7423926; doi:10.1038/s41467-020-17821-1)
Supplement: Supplementary file 6 — Reporting Summary [file 41467_2020_17821_MOESM6_ESM.pdf]

nature research

Corresponding author(s): Luca Scorrano

Last updated by author(s): Jul 7, 2020

Reporting Summary

Nature Research wishes to improve the reproducibility of the work that we publish. This form provides structure for consistency and transparency in reporting. For further information on Nature Research policies, see Authors & Referees and the Editorial Policy Checklist.

Statistics

For all statistical analyses, confirm that the following items are present in the figure legend, table legend, main text, or Methods section.

n/a	Confirmed	
☐	☒	The exact sample size (*n*) for each experimental group/condition, given as a discrete number and unit of measurement
☐	☒	A statement on whether measurements were taken from distinct samples or whether the same sample was measured repeatedly
☐	☒	The statistical test(s) used AND whether they are one- or two-sided<br>*Only common tests should be described solely by name; describe more complex techniques in the Methods section.*
☒	☐	A description of all covariates tested
☐	☒	A description of any assumptions or corrections, such as tests of normality and adjustment for multiple comparisons
☐	☒	A full description of the statistical parameters including central tendency (e.g. means) or other basic estimates (e.g. regression coefficient) AND variation (e.g. standard deviation) or associated estimates of uncertainty (e.g. confidence intervals)
☐	☒	For null hypothesis testing, the test statistic (e.g. *F*, *t*, *r*) with confidence intervals, effect sizes, degrees of freedom and *P* value noted<br>*Give P values as exact values whenever suitable.*
☒	☐	For Bayesian analysis, information on the choice of priors and Markov chain Monte Carlo settings
☒	☐	For hierarchical and complex designs, identification of the appropriate level for tests and full reporting of outcomes
☒	☐	Estimates of effect sizes (e.g. Cohen's *d*, Pearson's *r*), indicating how they were calculated

Our web collection on statistics for biologists contains articles on many of the points above.

Software and code

Policy information about availability of computer code

Data collection	LasAF 4.0 (Leica), ZenLite 2.1 blue edition (Zeiss), FACS Diva v8.0.1 (BD) Software
Data analysis	ImageJ 1.51 (NIH); Origin 9 (Origin Lab); GraphPad Prism 8.3.1 (Graphpad).

For manuscripts utilizing custom algorithms or software that are central to the research but not yet described in published literature, software must be made available to editors/reviewers. We strongly encourage code deposition in a community repository (e.g. GitHub). See the Nature Research guidelines for submitting code & software for further information.

Data

Policy information about availability of data

All manuscripts must include a data availability statement. This statement should provide the following information, where applicable:

- Accession codes, unique identifiers, or web links for publicly available datasets
- A list of figures that have associated raw data
- A description of any restrictions on data availability

The Source Data underlying the following Figures: 1b, c, e, f; 2b, d, e, g; 3b, c, e, f, g,; 4b, d, f, h, j, k; 5a, c, e, f, g; and Supplementary Figures: 1b, d, f; 2b, d, f; 3b; 4b, c, d; 5b, d, e-m are provided in the Source Data File. All data are available from the corresponding author upon reasonable request. Source data are provided with this paper.

October 2018

Field-specific reporting

Please select the one below that is the best fit for your research. If you are not sure, read the appropriate sections before making your selection.

☒ Life sciences ☐ Behavioural & social sciences ☐ Ecological, evolutionary & environmental sciences

For a reference copy of the document with all sections, see nature.com/documents/nr-reporting-summary-flat.pdf

Life sciences study design

All studies must disclose on these points even when the disclosure is negative.

Sample size

For in vivo experiments, sample size was predetermined by using power analysis and the following formula n=3 (za + zb)2 (s/D)2 where n=number of animals analyzed; a=0.05 (P, predetermined) za = 1.96; b=20% (error factor, predetermined) zb= 0.80; s=10% (variance), determined on the basis of experience and literature; D=20% (significant difference to obtain) determined on the basis of experience and literature ; 3=technical repeats to exclude artifacts

Data exclusions

No data were excluded.

Replication

Experiments were replicated by different investigators belonging to the lab of NT (C. Elegans), LS (imaging and mouse experiments). Number of independent replicates of each panel are described in the legends. All attempts of replication were successful.

Randomization

Mice could not be randomized because different genetic backgrounds were analyzed. Primary RGCs of different genetic backgrounds could not be randomized. Primary RGCs were randomized to pharmacological treatments with Rapamycin/Bafilomycin and to tranfection with different plasmids.

Blinding

experimenters were always blinded to the identity of the sample.

Reporting for specific materials, systems and methods

We require information from authors about some types of materials, experimental systems and methods used in many studies. Here, indicate whether each material, system or method listed is relevant to your study. If you are not sure if a list item applies to your research, read the appropriate section before selecting a response.

Materials & experimental systems

n/a	Involved in the study	
☐	☒	Antibodies
☒	☐	Eukaryotic cell lines
☒	☐	Palaeontology
☐	☒	Animals and other organisms
☒	☐	Human research participants
☒	☐	Clinical data

Methods

n/a	Involved in the study	
☒	☐	ChIP-seq
☐	☒	Flow cytometry
☒	☐	MRI-based neuroimaging

Antibodies

Antibodies used

Atg7 (Sigma, cat. A2856, lot. 094M808V), GFP (ThermoScientific cat. A11120, lot. 1037264), GFP (Invitrogen, cat. A11122, clone 3E6, lot. 1711553), LC3 (Nanotools, cat. 0231-100/LC3-5F10, clone 5F10, lot. 0231S0302), LC3 (Cell Signalling, cat. 2775, lot. 10), p62/SQSTM1 (Sigma, cat. P0067, lot. 103M4785V), Phospho-AMPKα (Thr172) (Cell Signalling, cat. 2535, clone 40H9, lot. 16), Ulk1 (Novus Biologicals, cat. NBP2-41217, lot. 7335-1203), β-Tubulin III isotype III (Sigma, cat. T8660, clone SDL.3D10, lot. 110M4837), OPA1 was from Dr. A. van der Bliek (University of California, Los Angeles, USA), Goat anti-Mouse IgG (H+L) Cross-Adsorbed Secondary Antibody, Alexa Fluor 488 (Invitrogen, cat. A-11001, lot. 1110070), Goat anti-Rabbit IgG (H+L) Cross-Adsorbed Secondary Antibody, Alexa Fluor 488 (Invitrogen, cat. A-11008, lot. 1069855), Goat anti-Mouse IgG (H+L) Cross-Adsorbed Secondary Antibody, Alexa Fluor 568 (Invitrogen, cat. A-11004, lot. 1069849), Goat anti-Rabbit IgG (H+L) Cross-Adsorbed Secondary Antibody, Alexa Fluor 488 (Invitrogen, cat. A-11011, lot. 1073082), Goat anti-Mouse IgG (H+L) Cross-Adsorbed Secondary Antibody, Alexa Fluor 405 (Invitrogen, cat. A-31553, lot. 1126599), Goat anti-Rabbit IgG (H+L) Cross-Adsorbed Secondary Antibody, Alexa Fluor 405 (Invitrogen, cat. A-31556, lot. 1010119).

Validation

Antibodies used in this study were commercially available (except OPA1 antibody). Fixation, permeabilization and incubation were optimized to obtain the higher signal-to-noise ratio for each antibody. OPA1 antibody has been validated in Griparic et al., JBC, 2004. The following statements about species reactivity are found on the websites of the commercial antibodies used. Atg7 (Sigma) "Rabbit anti-ATG7 antibody was used for immunohistochemistry on sections of mouse midbrain fixed in 4% paraformaldehyde at 1:2000."; GFP (ThermoScientific) "Immunofluorescence (IF) View 129 publications"; LC3 (Nanotools) "Species Reactivity: Human Mouse Rat Dog Hamster yes yes yes yes yes"; LC3 (Cell Signaling) "Supporting Data REACTIVITY H M R SENSITIVITY Endogenous"; p62/SQSTM1 (Sigma) "Specificity: Anti-p62/SQSTM1 recognizes human, rat, and mouse p62/SQSTM1. The antibody may be used in various immunochemical techniques including immunoblotting (~ 62 kDa), immunoprecipitation,

and indirect immunofluorescence."; Phospho-AMPKα (Thr172) (Cell Signalling) "Supporting Data: REACTIVITY H M R Hm Mk Dm Sc; SENSITIVITY Endogenous"; Ulk1 (Novus Biologicals) "Reactivity Hu, Mu, Rt; Applications WB, ELISA, IHC, IHC-P, ICC/IF"; β-Tubulin III isotype III (Sigma) "species reactivity bovine, boar, rat, human; enhanced validation knockout".

Animals and other organisms

Policy information about studies involving animals; ARRIVE guidelines recommended for reporting animal research

Laboratory animals	1) mouse, Opa1flx/flx (Cogliati et al., 2013), male, from 3 to 12 months;<br>2) mouse, Atg7flx/flx (Komatsu et al., 2008), male, from 0 to 2 post-natal days and from 3 to 12 months;<br>3) mouse, Gt(ROSA)26Sor+/lox-Stop-lox-mito-YFP(Sterky et al.,2011), male, 4 months;<br>4) mouse, C57BL/6-Tg(Grik4-cre)G32-4Stl/J (Jackson laboratories), male,from 3 to 12 months;<br>5) mouse, C57BL/6J (Jackson laboratories), from 0 to 2 post-natal days and from 3 to 12 months.
Wild animals	The study did not involve wild animals.
Field-collected samples	The study did not involve samples collected from the field.
Ethics oversight	All mouse procedures were performed according to approved protocols (protocol 32/2011 CEASA University of Padua and 318/2015 Italian Ministry of Health).

Note that full information on the approval of the study protocol must also be provided in the manuscript.

Flow Cytometry

Plots

Confirm that:

☒ The axis labels state the marker and fluorochrome used (e.g. CD4-FITC).

☒ The axis scales are clearly visible. Include numbers along axes only for bottom left plot of group (a 'group' is an analysis of identical markers).

☒ All plots are contour plots with outliers or pseudocolor plots.

☒ A numerical value for number of cells or percentage (with statistics) is provided.

Methodology

Sample preparation	Retinal cells from 3-months old mice were dissociated as previously described. For the evaluation of YFP purity as RGCs, cells were fixed with 3.7% paraformaldehyde for 30 minutes at 4°C, permeabilized and blocked for 1 hour with Triton-X-100 0.1% and BSA 5%, incubated for 1 hour with Brn3a (Abcam) and GFP (Invitrogen) antibodies and 30 minutes with Alexa-fluor 594 and 405 (Invitrogen). For autophagy flux experiments, retinal cells were treated with rapamicyn and bafilomicyn A. Then cells were fixed and stained with LC3 (Cell Signalling) and GFP antibodies following the above protocol.
Instrument	BD FACS Canto II.
Software	BD FACS Diva Software.
Cell population abundance	For autophagic flux analysis, LC3+YFP-405+ cells were ~700; for RGCs purity, Brn3a+YFP-405+ cells were ~600.
Gating strategy	In all experiments, cells were gated using FSC-A vs SSC-A by size and granularity. YFP+ and Brn3a+ or LC3+ cells were identified by excluding the positivity of cells marked solely with secondary antibodies.

☒ Tick this box to confirm that a figure exemplifying the gating strategy is provided in the Supplementary Information.
