# Supplementary material for: Inhibition of autophagy curtails visual loss in a model of autosomal dominant optic atrophy
Source: Nat Commun. 2020 Aug 12;11:4029. doi: 10.1038/s41467-020-17821-1 (PMC7423926; doi:10.1038/s41467-020-17821-1)
Supplement: Supplementary file 2 — Peer Review File [file 41467_2020_17821_MOESM2_ESM.docx]

Peer Review Information

**Journal:** Nature Communications

**Manuscript title:** Inhibition of autophagy curtails visual loss in a model of autosomal dominant optic atrophy

**Corresponding author name(s):** Dr Luca Scorrano

**Editorial notes:**

**Transferred manuscripts**

This manuscript has been previously reviewed at another journal that is not operating a transparent peer review scheme. This document only contains reviewer comments and rebuttal letters for versions considered at *Nature Communications*.

**Reviewer comments & decisions:**

**Reviewer comments, first version:**

Reviewer #1 (Remarks to the Author):

The work is not convincing mainly due to technical aspects.
- Colocalization analysis is apparently done in 3D reconstructions of stacks of confocal images, thus it is impossible to distinguish real colocalization (the two colours overlap in one z-plane) from overlapping colours in two different planes. If colocalization is done in individual z planes it should be mentioned.
- The author’s assume that the density and length of mitochondria are independent to the distance to the soma and they take images at different locations. If it is not an assumption it should be mentioned or taken into account.
- Statistical analysis is done only with parametric tests and no normality or equal variances tests are performed, again if they are performed they have not been mentioned.
- The expression of the OPA mutations induces cell death, so it is possible that all the effects are quantified in dying cells? This could influence mitochondria fluorescence. At 24h there is 40% of TUNEL positive cells (Figure 2f). Also wt cells are missing and a picture showing the real TUNEL staining should be shown.
- What is the rationale behind using a specific subtype of retinal ganglion cells (Grik4-Cre) instead of a more general retinal ganglion cell type? This would be more disease relevant as all retinal cells are mutated in patients.
- How is the hillock identified in the cryosection in Figure 5e (second e). How do they identify the retinal ganglion cells? there are also other cells in the layer as the displacled amacrine cells. What is the white line?
- In Figure 5 why they do stain mitochondria with OPA instead of a more general mitochondrial marker? Does the antibody recognize the mutated protein?
- Why are the autophagosome not evenly distributed in the OPA1 negative retinal ganglion cells (line 214), what does this mean? Why is this important?
- What happens to AMPk and ULk1 localization in the Atg7 deficient cells? Does it change?

Minor points:
- Figure 2a, CCCP as stated in the legend or FCCP as in the figure?.
- The manuscript is difficult to follow with very long and complicated sentences. It also lacks depth for concepts, results and discussion. For example why do they use these three different OPA1 mutations?
- References are missing in the introduction. What is the relevance for the human disease?. The mix mouse and human facts in lines 55 to 57, making it unclear for the reader.
- Figures are complicated, for example they mix autophagsomes and mitochondria in the same graph, for example Figure 1e.
- There are two e in the legend of figure 5.

**Author rebuttal, first version:**

**Reviewer #1 (Remarks to the Author):**

The work is not convincing mainly due to technical aspects. Colocalization analysis is apparently done in 3D reconstructions of stacks of confocal images, thus it is impossible to distinguish real colocalization (the two colours overlap in one z-plane) from overlapping colours in two different planes. If colocalization is done in individual z planes it should be mentioned.

We apologize for the missing information. Colocalization was measured on individual z-planes. This information has been included in the paragraph" Methods-Imaging": "*Colocalization between autophagosomes and mitochondria was quantified using Manders’ coefficient on individual z-planes of stacks of confocal images*".

The author’s assume that the density and length of mitochondria are independent to the distance to the soma and they take images at different locations. If it is not an assumption it should be mentioned or taken into account.

Thank you for pointing this out. We always measured number and length of mitochondria in the whole axon. In particular, the density of mitochondria was calculated as the total number of mitochondria found in the axon, divided by the total length of the said axon. We boxed randomly selected areas of axons to magnify representative mitochondria as an example. We corrected the explanation of the method of calculation in the paragraph “Methods-Imaging”:

"*Length of mitochondria and axons were manually traced. Length and number of mitochondria were quantified using the Multimeasure plug-in of Image J. All mitochondria retrieved in entire axon length were considered for the analysis*".

Number or morphology of mitochondria did not depend on their distance from the soma. Indeed, in ADOA mutants RGCs most if not all mitochondria are punctuated. The few mitochondria retrieved in the axon were found at different distance from the soma. Let us look at the case of Opa1^K301A^ expressing RGCs as an example: in Fig.1a there is only one mitochondrion retrieved in the central part of the axon; in Fig.1b there are 2 mitochondria, both very close to the soma. All these mitochondria are short and round. We added a statement to highlight this observation in the section “Results-Localized mitophagy in proximity of the axonal hillock of ADOA RGCs”:

"*Interestingly, axons of pathogenic mutants were also depleted of mitochondria (Fig.1a,c). This observation was similar to what is shown in dendrites of cortical neurons and in Cre-transduced Opa1^fl/fl^ RGCs (Supplementary Fig.1c,d). (...) Moreover, fragmented mitochondria appeared at different distances from the soma (Fig.1a,c)*".

Statistical analysis is done only with parametric tests and no normality or equal variances tests are performed, again if they are performed they have not been mentioned.

We thank the referee for pointing this out. We included the information requested in the "Methods-Statistical analysis" section:

"*Statistical significance was calculated by one-way or two-way ANOVA test with Bonferroni mean comparison between the indicated samples. Nonparametric ANOVA test were applied if assumptions of normality and homoscedasticity were not respected*".

The expression of the OPA mutations induces cell death, so it is possible that all the effects are quantified in dying cells? This could influence mitochondria fluorescence.

First, we always discarded RGCs that displayed a dying cell morphology (shrinkage, axonal retraction, blebbing…) from the analysis of mitochondrial fragmentation and autophagosome accumulation.

Second, we verified if fluorescence of the mitochondrial markers used here was affected by mutant Opa1 expression. We therefore fixed RGCs and immunostained mitochondria with endogenous TOM20 (green) in RGCs overexpressing Cherry-Tubulin (red). The density of mitochondria in the axon was superimposable to that observed when we labelled mitochondria with the fluorescent protein mtRFP (Fig. below). We thus reasoned that the fluorescence of mtRFP was indicative of mitochondria present and therefore we used this probe. We added this control in the Supplementary Fig.1e,f and we added the following integration in "Results-Localized mitophagy in proximity of the axonal hillock of ADOA RGCs": "*The observed reduction in axonal density of mitochondria was not a consequence of mtRFP fluorescence dimming. Indeed, we recorded a similar reduction in axonal mitochondrial density when we immunostained mitochondria using the outer mitochondrial membrane (OMM) marker TOM20 in Opa1^K301A^ RGCs (Supplementary Fig.1e,f)*".

**(a)** Representative z-projections of stacks of confocal images of Cherry-Tubulin (red) in primary RGCs co-transfected with the indicated plasmids and after 24 hrs fixed and immunostained with TOM20 (green). Bar, 20 μm.

**(b)** Quantification of mitochondrial axonal density in experiments as in (e). Data represent mean±SE of 3 independent experiments (n=20 cells/condition).*, p<0.05 in a non parametric one-way ANOVA test followed by Bonferroni post hoc test.

At 24h there is 40% of TUNEL positive cells (Figure 2f). Also wt cells are missing and a picture showing the real TUNEL staining should be shown.

Thank you for pointing this out. We measured apoptosis in wt cells overexpressing Opa1 mutants as requested. As expected from other published models, ADOA mutants induced apoptosis while Opa1 overexpression had no effect on cell death. We added these data and representative images in the revised manuscript. Specifically, we added pictures and quantification of TUNEL staining in EV and Cre expressing Atg7flx/+ RGCs transfected with Opa1^K301A^ in Fig.2f,g and we added a new Supplementary Figure to report the full experiment of TUNEL analysis in RGCs expressing all the plasmids used here (new Supplementary Fig.3).

What is the rationale behind using a specific subtype of retinal ganglion cells (Grik4-Cre) instead of a more general retinal ganglion cell type? This would be more disease relevant as all retinal cells are mutated in patients.

As the reviewer knows, other drivers promote expression of Cre also in other retinal cell types, whereas the Grik4-Cre line is unique in that it expresses Cre specifically and only in RGCs (Ivanova et al., Neuroscience, 2010). We indeed could confirm that Opa1 deletion occurred exclusively in RGCs by FACS analysis (Supplementary Fig.6a-d). We therefore decided to use this mouse strain to avoid in our *in vivo* tests any effect from other retinal cells. To take this useful comment into consideration we modified the paragraph “Results - Autophagy inhibition corrects visual loss of an RGC-specific Opa1 knockout mouse”:

"*We first decided to develop an ADOA mouse model based on conditional Opa1 ablation in RGCs. To this end we crossed Opa1^fl/fl^ with Grik4-Cre mice that express the Cre recombinase exclusively in RGCs, thus avoiding unwanted influence from deletion of Opa1 also in other retinal cells. Moreover, Grik4-positive RGCs represents a RGCs subpopulation sensitive to moving stimuli, i.e. the ones presented to the mice in the behavioural tests performed in our study*".

How is the hillock identified in the cryosection in Figure 5e (second e).

We did not mark the axonal hillock. We calculated the number of autophagosomes and the distribution of mitochondria and autophagosomes with respect to the soma. We analysed the symmetry of distribution of these organelles using skewness analysis in Image J. We added this information in "Methods-Imaging": "*The distribution of autophagosomes and mitochondria was measured on manually-traced somas in primary RGCs and retinal sections using the "skewness" analysis of ImageJ*". Furthermore, we also included this information in “Results-Autophagy inhibition corrects visual loss of an RGC-specific Opa1 knockout mouse”: "*To verify if the mouse model was mimicking the mitochondrial and autophagosomal phenotype observed in vitro, we analyzed the distribution of these organelles and the autophagic levels in ADOA mice. Immunostaining of retinal sections revealed that mitochondria and autophagosomes were distributed asymmetrically in the RGC layer of ADOA mice (Fig.5d,f)*".

What is the white line?

The white lines limit the cell bodies. We describe them more clearly in the "Figure legends": "*The soma of RGCs are circled with white lines in right p*anels".

How do they identify the retinal ganglion cells? there are also other cells in the layer as the displacled amacrine cells.

From our FACS analysis (Supplementary Fig.6a-d), we excluded the presence of other retinal cells targeted by the Cre recombinase. We also noticed that ~70% of RGCs were not targeted by Grik-Cre (Supplementary Fig.6a), as expected from another publication (Ivanova et al., Neuroscience, 2010). We reasoned that if we used a general marker to identify RGCs, we would have included also untargeted RGCs. Therefore, we selected the subgroup of Opa1-depleted RGCs that were less stained by the OPA1 antibody (see below). We acknowledge that this limits the correct identification of RGCs in wt animals, but we analysed all the cells in the ganglion cell layer and we never measured aggregated mitochondria or autophagosomes in WT samples. We thank the referee for this good point. We wrote in the revised version of the manuscript that all cells of the ganglion cell layer were considered in the analysis of wt animals in the "Methods-Imaging" section: "*Opa1^ΔRGC/ΔRGC^ RGCs were identified by reduced staining of OPA1 in ADOA mice while all cells of the ganglion cell layer were analyzed in WT mice*".

In Figure 5 why they do stain mitochondria with OPA instead of a more general mitochondrial marker? Does the antibody recognize the mutated protein?

We used the antibody produced in the laboratory of A. Van der Bliek (Griparic et al., JBC, 2004). This antibody recognizes a C-terminal sequence of Opa1 (residues 616–850). Van Der Bliek and colleagues used this antibody to immunostain cells overexpressing the K301A mutant. The signal of the OPA1 antibody colocalized with that of an immunostaining for ATP synthase and with that of MitoTracker (Fig. 1c,4a of Griparic et al.). We therefore reasoned that this antibody might mark mitochondria in retinal cryosections. Indeed, the signal of OPA1 colocalizes with that of the mitochondrial marker Cyclophilin D (Fig. below, picture acquired in the ganglion cell layer) and it is also less intense in Opa1-depleted RGCs.

*Staining of OPA1 (red) and mitochondria (CYPD, cyclophilin D, green) in the ganglion cell layer of WT and Opa1-depleted RGCs (Opa1^ΔRGC/ΔRGC^).*

Why are the autophagosome not evenly distributed in the OPA1 negative retinal ganglion cells (line 214), what does this mean? Why is this important?

The mouse model allowed us to verify if inhibition of autophagy could have a beneficial effect on Opa1-deficient RGCs also *in vivo*. Indeed, when autophagy is inhibited visual impairment is curtailed in ADOA mice. In addition, we report that we could observe the phenotypes in primary ADOA RGCs also *in vivo*, in the ADOA mouse model. In our opinion, the observation of increased autophagy and asymmetric distribution of autophagosomes *in vivo* in the Opa1-depleted RGCs supports the importance of altered autophagy in the pathogenesis of ADOA.

We believe that this is important because we think that compartmentalized activation of autophagy and autophagosome accumulation is a hallmark of the disease. The initiator of autophagy AMPK (or better, its phosphorylated active form) and its effector ULK1 are localized in the same region of RGCs where we find the autophagosomes. It is reasonable to think that the AMPK-ULK1 axis could promote autophagosomes accumulation in this region. Indeed, in primary RGCs the number of autophagosomes is reduced when AMPK is genetically blocked.

We modified the text taking into account the observation of the Referee in "Results-Autophagy inhibition corrects visual loss of an RGC-specific Opa1 knockout mouse": "*To verify if the mouse model was mimicking the mitochondrial and autophagosomal phenotype observed in vitro, we measured the levels of LC3^+^ vesicles and the distribution of these organelles and of mitochondria in Opa1-deleted RGCs in vivo. Immunostaining of retinal sections revealed that mitochondria and autophagosomes were distributed asymmetrically in Opa1-deleted RGCs in mice (Fig.5d,f). Furthermore, the abundance of LC3+ puncta per cell was doubled in retinal sections of ADOA mice (Fig.5d,e) and the intensity of LC3B fluorescence was also doubled in sorted mtYFP+ RGCs by FACS (Fig.5g). These data indicate that like in RGCs expressing Opa1 mutants, autophagosomes accumulate and mitochondria and autophagosomes are asymmetrically distributed in Opa1-deficient RGCs in vivo* ".

What happens to AMPk and ULk1 localization in the Atg7 deficient cells? Does it change?

We did not perform this experiment. While this experiment constitutes a very interesting follow up to understand if ATG7 can influence AMPK and ULK1 localization in ADOA, we think that at this moment it does not add to the extensive list of controls on autophagy-dependent modulation of the ADOA phenotype already reported in the paper.

Minor points:

Figure 2a, CCCP as stated in the legend or FCCP as in the figure?.

Thank you for pointing this out. We used CCCP. We corrected the figure.

The manuscript is difficult to follow with very long and complicated sentences. It also lacks depth for concepts, results and discussion. For example why do they use these three different OPA1 mutations?

We understand the concerns of the Referee and we revised the text. For example, we justified the use of three different Opa1 mutations in "Results-Localized mitophagy in proximity of the axonal hillock of ADOA RGCs": "*Therefore, we wanted to specifically inspect the effect of mutated Opa1 in RGCs, the cell type primarily affected by the disease. Because the GTPase and the coiled-coil domains of OPA1 are hotspots for ADOA mutations, we used two pathogenic Opa1 mutants modeling frequent ADOA mutations in these two regions: Opa1^K301A^, a GTPase domain mutant that reduces Opa1 GTPase activity, and Opa1^R905*^, a truncative mutant of the coiled-coil domain important for Opa1 protein-protein interaction. In addition, as a control mutant we expressed Opa1^Q297V^, a constitutively active form of Opa1 that is not an ADOA mutant. Indeed, while Opa1^K301A^ and Opa1^R905*^ fragment mitochondria and induce Opa1-dependent apoptosis^4,28,30^, Opa1^Q297V^ shows opposite effects*".

References are missing in the introduction. What is the relevance for the human disease? The mix mouse and human facts in lines 55 to 57, making it unclear for the reader.

In the introduction, we wanted to create a link between human ADOA and ADOA mouse models. Our finding that inhibition of autophagy restores vision in an ADOA mouse model could offer the molecular basis to select some drugs to treat the disease, as we wrote in the Discussion: "*Because Atg7 depletion permanently protects from the visual loss caused by Opa1 deletion, we conclude that mitochondria with reduced Opa1 levels can nevertheless sustain RGC function throughout rodent life. RGCs constitute the innermost retinal cell layer and are hence directly exposed to the vitreous: intravitreal delivery of autophagy inhibitors, or of drugs modulating AMPK activity might offer a therapeutic strategy to curtail the visual loss in ADOA patients*".

We tried to separate mouse and human references in lines 55 to 57 of the Introduction (previous manuscript), in the following way: "*Autosomal dominant optic atrophy (ADOA) is an untreatable disease caused by Opa1 mutations. ADOA is clinically characterized by early childhood bilateral visual loss and the primarily cells affected by the disease in humans are RGCs. However, due to the limited accessibility of retinal material, the most informative data come from rodent studies. Changes in autophagy are prominent features of mouse models of ADOA*".

Finally, in the Introduction we preferred to cite reviews for broad topics as for mitochondrial dynamics, but we changed them with original articles.

Figures are complicated, for example they mix autophagsomes and mitochondria in the same graph, for example Figure 1e.

Unfortunately, the figures are already too data dense to provide two different graphs here. However, we clearly indicated which box plot refers to mitochondria and which to autophagosomes.

There are two e in the legend of figure 5.

We corrected the text.

**Reviewer comments, second version:**

Reviewer #1 (Remarks to the Author):

the author's have added some clarifications that have improved the paper

**Author rebuttal, second version:**

Reviewer #1 (Remarks to the Author):

the author's have added some clarifications that have improved the paper

Thank you for your positive feedback.

**Reviewer comments, third version:**

**Author rebuttal, third version:**
